# Qat use and esophageal cancer in Ethiopia: A pilot case-control study

**DOI:** 10.1371/journal.pone.0178911

**Published:** 2017-06-08

**Authors:** Maria E. Leon, Mathewos Assefa, Endale Kassa, Abate Bane, Tufa Gemechu, Yared Tilahun, Nigatu Endalafer, Gilles Ferro, Kurt Straif, Elizabeth Ward, Abraham Aseffa, Joachim Schüz, Ahmedin Jemal

**Affiliations:** 1 Section of Environment and Radiation, International Agency for Research on Cancer (IARC), Lyon, France; 2 Department of Internal Medicine, Faculty of Medicine, Addis Ababa University, Addis Ababa, Ethiopia; 3 Pathology, Faculty of Medicine, Addis Ababa University, Addis Ababa, Ethiopia; 4 Armauer Hansen Research Institute (AHRI), Addis Ababa, Ethiopia; 5 Section of IARC Monographs, International Agency for Research on Cancer (IARC), Lyon, France; 6 Surveillance and Health Services Research, American Cancer Society (ACS), Atlanta, United States of America; University Hospital Llandough, UNITED KINGDOM

## Abstract

**Background:**

Qat (*Catha edulis*) chewing is reported to induce lesions in the buccal mucosa, irritation of the esophagus, and esophageal reflux. Case series suggest a possible etiological role in oral and esophageal cancers. This pilot study aimed to generate preliminary estimates of the magnitude and direction of the association between qat use and esophageal cancer (EC) risk and to inform the logistics required to conduct a multi-center case–control study.

**Methods:**

Between May 2012 and May 2013, 73 EC cases (including 12 gastro-esophageal junction cases) and 133 controls matched individually on sex, age, and residence were enrolled at two endoscopy clinics and a cancer treatment hospital in Addis Ababa. A face-to-face structured questionnaire was administered. Qat use was defined as ever having chewed qat once a week or more frequently for at least one year. Odds ratios were calculated using conditional logistic regression.

**Results:**

Only 8% of cases resided in Addis Ababa. Qat use was more frequent in cases (36%) than in controls (26%). A 2-fold elevation in EC risk was observed in ever qat chewers compared with never users in unadjusted conditional logistic regression (OR = 2.12; 95% CI = 0.94, 4.74), an association that disappeared after adjusting for differences in tobacco use, consumption of alcohol and green vegetables, education level, and religion (OR = 0.95; 0.22, 4.22). Among never tobacco users, however, a non-significant increase in EC risk was suggested in ever qat users also after adjustment. Increases in EC risk were observed with ever tobacco use, alcohol consumption, low consumption of green vegetables, a salty diet, illiteracy, and among Muslims; the four latter associations were significant.

**Conclusions:**

This pilot study generated EC risk estimates in association with a habit practiced by millions of people and never before studied in a case–control design. Results must be interpreted cautiously in light of possible selection bias, with some demographics such as education level and religion differing between cases and controls. A large case–control study with enrolment of EC cases and carefully matched controls at health facilities from high-risk areas in the countryside, where the majority of cases occur, is needed to further investigate the association between qat use and EC.

## Introduction

The masticated leaves of the alkaloid-rich non-nicotine-containing tree-like shrub Qat (chat or khat; *Catha edulis* Forsskal) [1] induce a temporary stimulant effect. The psychoactive component, cathinone, produces a similar effect to amphetamine, but milder [2]. It is estimated that qat chewing is a habit of millions of people in their everyday lives in several countries in the Arabian Peninsula and eastern and southern Africa [3,4].

In Ethiopia, the world’s largest producer of high-quality qat, qat chewing is widespread and is considered to be a tradition in the Muslim community, particularly in men [4–6]. Over the past several decades, qat use has become more common in all sociodemographic groups. However, it remains more prevalent in Muslims than in Christians, in men than in women, in more educated than in less educated people, and in middle-aged people than in younger or older people; prevalence of qat use is similar in urban and rural areas [7,8]. Based on a large cross-sectional survey conducted in 2006 in Addis Ababa, in which more than 93% of the participants were Christians, the prevalence of qat use was about 18% in men and 2% in women [9]. This contrasts with a prevalence of more than 50% reported in Butajira, a city about 130 km south of Addis Ababa, where more than 70% of the population is Muslim [10]. Of the men who reported regular qat chewing in 2006 in Addis Ababa, 40% were also current smokers and 20% were binge drinkers [9]. According to the most recently available national survey, from 2011, 28% of men and 11% of women reported ever having chewed qat [7].

The majority of the alkaloid content of qat is extracted into saliva, and most of it is absorbed through the oral mucosa [11]. Previous studies, all conducted outside of Ethiopia, have associated long-time qat chewing with adverse health conditions in the upper digestive tract (UDT), including esophagitis, gastritis, gastro-esophageal reflux, and other digestive disturbances [12,13]. Cases of oral cancer and of esophageal cancer have also been reported both in exclusive qat chewers and in concurrent users of qat and tobacco products [14–17]. Furthermore, exclusive qat chewing has also been associated with genetic damage measured by the micronucleus test in oral cells [18]. However, no well-designed analytical epidemiological studies have yet been conducted to provide evidence about whether qat use is a risk factor for the occurrence of esophageal cancer [19].

In this paper, we report the results of a pilot case–control study on the association between esophageal cancer (EC) and qat use in Ethiopia, a first-ever description of a relative risk estimate for this association. The pilot study also investigated other suspected local practices as well as established EC risk factors. We also report lessons learned during the implementation of the pilot study that will inform the design of a subsequent large-scale epidemiological study.

## Methods

### Pilot study

The main goal of this pilot case–control study was to gather data needed to initiate a future large-scale case–control study to examine whether qat use, a deeply rooted social and cultural tradition in Ethiopia, is associated with risk of oral cancer and EC. Initially, the pilot study enrolled cases of oral cavity, pharyngeal, esophageal, and gastro-esophageal junction cancer, but here we report only the results for esophageal and gastro-esophageal junction cancers, because a substantial proportion of the enrolled cases of oral cavity and pharyngeal cancer turned out to be prevalent rather than incident cases. Due to sparse representative data on the annual number of EC cases diagnosed in Addis Ababa and in Ethiopia, one of the objectives of the pilot was to find out how many cases one can recruit over a certain time period at the planned recruitment sites. Therefore, no formal sample size calculation was made before this pilot case–control study was conducted.

### Recruitment

Ascertainment of study participants took place in three health facilities in Addis Ababa. Cancer patients were enrolled in the Oncology Department of the Tikur Ambessa Hospital (TAH), the only hospital in the country that offered radiotherapy during the study period, and in two private clinics that offer endoscopy services, the Mexico Higher Clinic (MHC) and the Adera Higher Clinic (AHC). Hospital- and visitor-type controls were recruited from other departments of the TAH. Enrolment of cancer cases started in May 2012 and ended in December 2012, whereas enrolment of controls started in August 2012 and ended in May 2013.

### Eligibility

#### Cases

Adults (≥ 18 years old) with a strong clinical suspicion (solid and/or liquid dysphagia and weight loss) at selected anatomical cancer sites or with confirmed incident primary EC or gastro-esophageal junction cancer according to the International Classification of Diseases for Oncology, 3rd edition, identified at the participating clinics and hospital, were eligible. Patients with a second or relapse EC and those who were mentally, emotionally, or physically unable to give consent were excluded. Eligible topography sites were: cervical esophagus (C15.0), upper third of the esophagus (C15.3), middle third of the esophagus (C15.4), lower third of the esophagus (C15.5), overlapping lesion of esophagus (C15.8), esophagus unspecified (C15.9), and cardia, NOS (cardio-esophageal junction, esogastric junction, gastro-esophageal junction) (C16.0).

For patients enrolled at the endoscopy clinics, the initial diagnosis was based on clinical suspicion, but a final diagnosis relied on histological or cytological confirmation. In patients consenting to donate biopsy specimens at endoscopy, three samples were taken and used to generate two formalin-fixed, paraffin-embedded (FFPE) blocks, and diagnosis slides, and the third sample for fresh tissue preservation. A majority of patients recruited at TAH were referred from other hospitals for treatment and did not have tumor tissue archived at TAH. For these patients, the diagnosis was based on histology slides or FFPE blocks, if these materials were available from the referral hospitals and patients consented to release of these specimens, or on a copy of the pathology report from the referral hospitals.

#### Controls

Two types of controls were included in the pilot study: (i) hospitalized, cancer-free patients with diagnoses not related to qat, tobacco, or alcohol from the inpatient or outpatient wards at TAH; and (ii) healthy people visiting inpatients at TAH and not related by blood to study cases or controls (as a sample of the healthy general population that presumably goes through similar mechanisms of selection as cases to reach the hospital). An attempt was made to enroll two controls (one of each type) per eligible case, and the case–control ratio achieved was 1:1.8. Controls were matched to cases on sex, age (± 5 years), and place of residence at least at the “zone” level (S1 Fig). Zone was considered the smallest geographical demarcation feasible for use in matching cases and controls on residence.

### Questionnaire

The qat part of the study questionnaire was newly developed after obtaining information on qat and qat use from the literature, with additional input from researchers from Yemen and Ethiopia. Demographic and lifestyle factors other than qat chewing, and questions related to Ethiopian dietary traditions, were derived from a structured questionnaire previously used in an IARC study on head and neck cancer. The questionnaire was adapted to reflect local dietary items and artisanal alcoholic beverages commonly consumed in Ethiopia. Lifetime history of qat, tobacco (smoked and smokeless), and alcohol use, diet, oral health, family history of cancer, and residential and occupational history were also covered. The original questionnaire in English was translated into Amharic and back-translated into English, to assess the accuracy of the Amharic version (the English (S1 File) and Amharic (S2 File) versions of the questionnaire are available as Supporting Information). The study questionnaire was pilot tested in 13 subjects (7 cancer cases and 6 healthy visitor-type controls) before initiation of the study, to determine (i) how easy it was to approach the subjects and obtain their consent to participate, (ii) comprehension, (iii) duration of the interview, and (iv) identification of unforeseen issues requiring modifications to the questionnaire. This preliminary testing did not constitute a validation of the questionnaire.

The structured questionnaire was read to study participants by study nurses using the Amharic version, and responses were recorded in the English version of the questionnaire in a face-to-face interview at the endoscopy clinics and the TAH. For study participants who were from the countryside and could not communicate in Amharic, the nurse, with the help of an interpreter if needed, explained questions in the language of the participant. Nurses received training on the administration of the questionnaire and study procedures before starting enrolment. To minimize the extent of missing data, the interviewer was allowed to probe the study participant.

### Consent procedure and ethics statement

Written informed consent was obtained from each participant included in the study. The pilot study was reviewed and approved by the following Institutional Review Boards: the IARC Ethics Committee in Lyon, France, the Morehouse School of Medicine in Atlanta, USA, and the Faculty of Medicine, Addis Ababa University, Ethiopia. Subjects were asked to consent or decline participation to each of the following study components: questionnaire administration (cases and controls), blood collection (cases and controls), mouthwash with gargle (cases and controls), tumor tissue or cytology slide collection (cases), and access to hospital records (cases and hospital controls).

### Data, variable definitions, and analysis

Data from each completed questionnaire were entered separately by two different data-entry clerks (thus entered twice) to rule out data entry errors.

Data collection aimed to assess lifetime qat use, including, but not limited to, ever and current use, frequency, duration, intensity, type of qat, typical time of retention in the mouth, swallowing of the saliva and/or plant residues, cessation, and length of abstinence. Qat use was defined as ever having chewed qat once a week or more frequently for at least one year. Cigarette smoking was defined as ever having smoked at least 100 cigarettes during the lifetime. Pipe smoking was defined as ever having smoked at least 50 pipes. Shisha smoking was defined as having smoked shisha once a week for at least one year. Smokeless tobacco use was defined as having chewed tobacco for at least one year. Thus, ever tobacco use refers to having used any of the above-listed tobacco products. Alcohol use was defined as ever having drunk *tela* (a fermented beverage made from cereals and *gešo*, a shrub of the Rhamnaceae family), *tej* (a brewed beverage made from fermented honey and *gešo*), *aräqe* (a distilled product made from fermented cereals, including wheat, maize, and sorghum, with higher alcohol content than *tela* or *tej*), beer, or hard liquor at least once a week for more than six months. The study questionnaire included a series of questions related to diet, asking participants to recall a year before the interview in controls and a year before the endoscopy or diagnosis in cases, including questions about: the staple food consumed at home, consumption of porridge or *genfo*, and frequency of consumption of green vegetables, other vegetables, and fruits. Smokiness inside the home was assessed in the residential history obtained.

Odds ratios and corresponding 95% confidence intervals were obtained using conditional logistic regression keeping the matched sets. To assess the risk of EC in users of both qat and tobacco, odds ratios for qat in ever tobacco users were contrasted with those in never tobacco users. Potential confounding by other known or suspected risk factors (e.g. alcohol use and dietary deficiencies) was also assessed. All analyses were conducted using Stata 12 (StataCorp LP, College Station, Texas). Results reported here are from combining the two sets of controls (inpatients and healthy controls), but in sensitivity analyses using only one of the control groups in each relative risk estimate, we checked for the main risk factors whether odds ratios differed by control group, due to the fact that as numbers became smaller, the confidence intervals of the two analyses usually overlapped widely.

## Results

**Fig 1:**
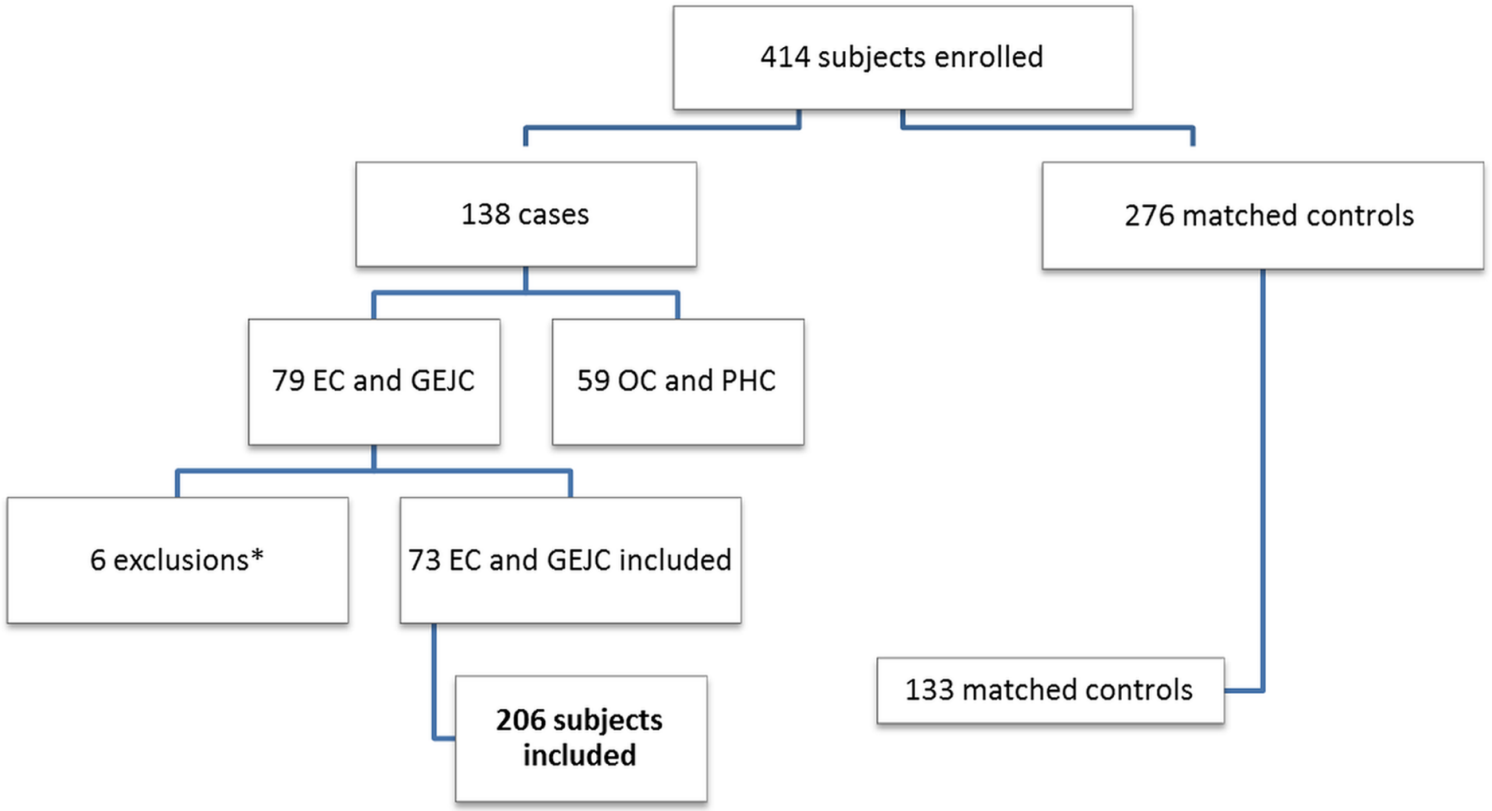
Number of cancer cases and matched controls included in the analysis. EC: esophageal cancer; GEJC: Gastro-esophageal junction cancer; OC: oral cancer; PHC: pharyngeal cancer. *Exclusions: 2 stomach cancer cases, 3 non-primary esophageal cancer cases (relapse), 1 eligible EC case refusing to complete the study questionnaire.

The numbers of EC cases and controls enrolled in the pilot study, showing exclusions, are displayed in Fig 1. The final sample size for the analysis was 206 participants: 61 EC cases and 12 gastro-esophageal junction cancer cases, for a total of 73 cases, and their 133 matched controls. In three suspected EC cases (representing 4.1% of all cases), the two endoscopy tissue samples examined by histopathology did not confirm the presence of cancer, but the quality of the specimens was labelled as poor and the size of the biopsy was very small. Based on clinical presentation and endoscopic examination, these cases were retained in the analysis as presumed cancers. The majority of the cases were enrolled at the endoscopy clinics; only 15.1% of the cases were recruited at the hospital (TAH), where all the controls were enrolled (Table 1). The majority of inpatient controls were enrolled from the wards treating urogenital (35%) and orthopedic (22.5%) health problems. Cancer cases showed an almost even sex distribution (51% men). Less than half (41%) of all cases were 60 years or older (mean age, 55.85 ± 11.88 years; range, 29–89 years). At the time of enrolment in Addis Ababa, more cancer patients resided in the Oromia Region (56%) than in any other region of the country, and the Oromo ethnicity accounted for 47% of the total enrolled cancer patients. A greater proportion of cancer cases were Muslims (64%) than were Christians (36%); this was opposite in controls (29% Muslims and 68% Christians). Eight-eight percent of cases had no formal education or at most primary instruction; in controls, this proportion was smaller, at 59%. EC cases with squamous cell carcinoma were more frequent (70%) than those with adenocarcinoma (19%) (Table 1).

**Table 1:** Demographic and clinical description of study participants included in the analysis.

		Esophageal		Junction		Total		Inpatient		Healthy		Total	
		n = 61		n = 12		n = 73		n = 40		n = 93		n = 133	
Sex	Male	30	49%	7	58%	37	51%	21	53%	49	53%	70	53%
	Female	31	51%	5	42%	36	49%	19	47%	44	47%	63	47%
Age	<40	5	8%	1	8%	6	8%	1	3%	8	9%	9	7%
	40–49	12	20%	1	8%	13	18%	5	13%	19	20%	24	18%
	50–59	19	31%	5	42%	24	33%	20	50%	32	34%	52	39%
	≥60	25	41%	5	42%	30	41%	14	35%	34	37%	48	36%
Region	Addis Ababa	6	10%			6	8%	4	10%	5	5%	9	7%
	Oromiya	32	52%	9	75%	41	56%	20	50%	53	57%	73	55%
	SNNP	12	20%	2	17%	14	19%	5	13%	22	24%	27	20%
	Somali	8	13%	1	8%	9	12%	9	23%	7	8%	16	12%
	Amhara									3	3%	3	2%
	Afar	3	5%			3	4%	2	5%	3	3%	5	4%
Ethnicity	Oromo	28	46%	6	50%	34	47%	14	35%	37	40%	51	38%
	Amhara	8	13%	1	8%	9	12%	8	20%	25	27%	33	25%
	Gurage	6	10%	2	17%	8	11%	8	20%	10	11%	18	13%
	Somali	8	13%	1	8%	9	12%	5	13%	5	5%	10	8%
	Other	11	18%	2	17%	13	18%	5	13%	16	17%	21	16%
Language	Amharic	24	39%	3	25%	27	37%	28	70%	82	88%	110	83%
	Oromigna	21	34%	5	42%	26	36%	7	18%	5	5%	12	9%
	Guragigna	4	7%	1	8%	5	7%	1	2%	2	2%	3	2%
	Somaligna	6	10%	1	8%	7	10%	2	5%	2	2%	4	3%
	Other	4	7%	2	17%	6	8%	1	2%			1	1%
	Unknown	2	3%			2	3%	1	2%	2	2%	3	2%
Education	Illiterate	45	74%	7	58%	52	71%	20	50%	26	28%	46	35%
	Primary	10	16%	2	17%	12	16%	8	20%	24	26%	32	24%
	Jun/Sen/Uni	5	8%	3	25%	8	11%	12	30%	41	44%	53	40%
	Unknown	1	2%			1	1%			2	2%	2	1%
Religion	Muslim	38	62%	9	75%	47	64%	16	40%	23	25%	39	29%
	Christian	23	38%	3	25%	26	36%	23	58%	68	73%	91	68%
	Other/None									1	1%	1	1%
	Unknown							1	2%	1	1%	2	2%
Enrolment	TAH	10	16.4%	1	8.3%	11	15.1%	40	100%	93	100%	133	100%
	Clinic 1	32	52.5%	8	66.7%	40	54.8%						
	Clinic 2	19	31.1%	3	25.0%	22	30.1%						
Location tumor	Upper 3rd	5	8.2%			5	6.8%						
	Middle 3rd	21	34.4%			21	28.8%						
	Lower 3rd	25	41.0%			25	34.2%						
	Esophagus NOS	7	11.5%			7	9.6%						
	Jnction			12	100%	12	16.4%						
	Cancer not confirmed	3	4.90%			3	4.1%						
Type of tumor	Squamous cell	49	80.3%	2	16.7%	51	69.9%						
	Adenocarcinoma	5	8.2%	9	75.0%	14	19.2%						
	Mixed	2	3.3%	1	8.3%	3	4.1%						
	Other	2	3.3%			2	2.7%						
	Cancer not confirmed	3	4.9%			3	4.1%						
Clinical condition	Healthy									93	100%		
	Uro-genital							14	35%				
	Ortopaedics							9	22.5%				
	Intestinal							4	10%				
	Infection							4	10%				
	Goiter							2	5%				
	Hernia inguinal							2	5%				
	Other							5	12.5%				

A slightly larger proportion of cases (36%) than controls (26%) were ever qat users (S1 Table), whereas duration of use among consumers did not differ much between cases (mean, 30 years) and controls (mean, 31 years). Overall, very few qat users had used qat for less than 10 years. The median retention time of the qat wad in the mouth during a chewing session was 4 hours in both cases and in controls. A higher proportion of cases (11%) than of controls (2.3%) retained the qat wad in the mouth overnight. Smoking during qat chewing sessions was reported in 16.4% of cases and in 11.3% of controls. Reporting of ever alcohol use was slightly higher in controls (41%) than in cases (30%), possibly related to the religious affiliation (S2 Table). Although the overall distribution of reported consumed staple food items did not differ between cases and controls, a slightly higher proportion of cases (21.9%) than controls (12%) specified *kocho* as the staple food. *Kocho* (*qocho*) is a local flatbread made using the starch obtained from the trunk of the false banana plant (*Ensete ventricosum*), fermented for several months and subsequently cooked in a griddle. Consumption of very salty food was more commonly reported by cases (26%) than by controls (1.5%).

A non-significant 2-fold elevation in risk of EC was seen in ever qat chewers compared with never users in unadjusted conditional analysis (odds ratio [OR] = 2.12; 95% confidence interval [CI]: 0.94, 4.74)) (Table 2), although this association disappeared after accounting for differences in tobacco use, consumption of alcohol and green vegetables, education level, and religion (OR = 0.95; 95% CI: 0.22, 4.22). No clear trend was observed between duration of qat use and risk of EC (Table 2). Tobacco use was associated with a more than 2-fold increase in risk of EC (OR = 2.62; 95% CI: 1.16, 5.92), the association in the adjusted model showed a larger magnitude but a lower precision, becoming non-significant (OR = 3.31; 95% CI: 0.53, 20.62) (Table 2). We did not observe a clear trend in EC risk with duration of tobacco use (Table 2). In sensitivity analysis according to control type, we observed a statistically significant elevation in risk of EC associated with ever tobacco use when including healthy visitor-type controls (OR = 3.02; 95% CI: 1.04, 8.77; *p* = 0.04) but not when limiting to hospital controls (OR = 1.75; 95% CI: 0.56, 5.51; *p* = 0.34) in fully adjusted unconditional logistic regression models. The association of qat chewing and risk of EC was also evaluated after stratifying by tobacco use. The risk of EC in ever qat users was elevated and statistically significant among never tobacco users (OR = 5.81; 95% CI: 1.21, 27.9) but not among ever tobacco users (OR = 0.76; 95% CI: 0.12, 4.61) (Table 2) in univariate analysis. After adjusting for differences in consumption of alcohol and green vegetables, education level, and religion, the risk of EC in ever qat users remained elevated but with a very wide confidence interval including 1 (OR = 6.75; 95% CI: 0.48, 96). The *p*-value for the interaction term between ever qat use and ever tobacco use was 0.06.

**Table 2:** Qat and tobacco use and risk of esophageal and gastro-esophageal junction cancers combined in a pilot case–control study in Addis Ababa.

Tobacco and qat use	Number cases / controls^a^	Odds Ratio (OR)^b^	95% CI	OR adjusted (education, religion, alcohol, green vegetables, and tobacco—if applicable)	95% CI
**Qat use**					
Never	47 / 99	1.00	Reference	1.00	Reference
Ever	26 / 34	2.12	0.94, 4.74	0.95	0.22, 4.22
		p = 0.07		p = 0.95	
**Qat, Never Tobacco**					
Never	38 / 93	1.00	Reference	1.00	Reference
Ever	12 / 16	5.81	1.21, 27.91	6.75	0.48, 96.00
		p = 0.03		p = 0.16	
**Qat, Ever Tobacco**					
Never	9 / 6	1.00	Reference	1.00	Reference
Ever	14 / 18	0.76	0.12, 4.61	0.00	0.00, -
		p = 0.76		p = 1.00	
**Qat duration (years)**					
Never	47 / 99	1.00	Reference	1.00	Reference
< 30 years	14 / 13	3.17	1.13, 8.91	2.30	0.23, 23.34
30+ years	12 / 19	1.96	0.74, 5.18	1.29	0.23, 7.27
Unknown	0 /2				
		p = 0.98		p = 0.25	
**Tobacco use**					
Never	50 / 109	1.00	Reference	1.00	Reference
Ever	23 / 24	2.62	1.16, 5.92	3.31	0.53, 20.62
		p = 0.02		p = 0.20	
**Tobacco duration (years)**					
Never	50 / 109	1.00	Reference	1.00	Reference
< 30 years	6 / 12	1.3	0.42, 4.04	3.27	0.31, 35.10
30+ years	14 / 11	3.59	1.35, 9.53	2.93	0.30, 28.31
Unknown	3 / 1	8.64	0.79, 94.76	63.4	0.00, 1.3e10
		p = 0.02		p = 0.25	

^a^ Cases of esophageal cancer and gastro-esophageal junction pooled. Inpatient and visitor-type control pooled

^b^ Conditional logistic regression results shown

Ever alcohol use was associated with a reduction in risk of EC (OR = 0.45; 95% CI: 0.21, 0.96) according to the unadjusted risk estimate, but the odds ratio increased to 2.3 (95% CI: 0.32, 16.53) after adjusting for tobacco use, consumption of green vegetables, education level, and religion (Table 3). Staple foods reported to have been consumed one year before the interview, or one year before the endoscopy or diagnosis, were suggestive of an elevation in risk of EC. Subjects who specified *kocho* as the staple food had a statistically significantly elevated EC risk in univariate analysis only (Table 3). Low consumption of green vegetables was associated with an increased risk of EC, with subjects consuming green vegetables less than once a week (OR = 3.57; 95% CI: 1.31, 9.71) or not at all (OR = 12.47; 95% CI: 2.91, 53) having a substantially higher risk of EC compared with those with daily consumption. These results were corroborated after adjusting for confounders (Table 3). Saltiness of food and smokiness inside the home were associated with an increased risk of EC in unadjusted analysis, whereas in adjusted analysis an elevation in risk remained only for saltiness of food (Table 3).

**Table 3:** Education level, religion, dietary habits, and risk of esophageal and gastro-esophageal junction cancers combined in a pilot case–control study in Addis Ababa.

Demographic, lifestyle & dietary factors	Number cases / controls^a^	Odds Ratio^b^, unadjusted	95% CI	Odds Ratio^b^, adjusted (education, religion, alcohol, green vegetables, and tobacco—if applicable)	95% CI
**Education**					
Illiterate	52 / 46	1.00	Reference	1.00	Reference
Primary	12 / 32	0.17	0.06, 0.50	0.07	0.01, 0.45
Jun/Sen/Uni	8 / 53	0.06	0.02, 0.19	0.02	0.00, 0.23
Unknown	1 / 2				
		p = 0.00		p = 0.00	
**Religion**					
Muslim	47 / 39	1.00	Reference	1.00	Reference
Christian	26 / 91	0.09	0.03, 0.26	0.05	0.01, 0.44
Other	0 / 1				
Unknown	0 / 2				
		p = 0.00		p = 0.01	
**Ethnicity**					
Oromo	34 / 51	1.00	Reference		Reference
Amhara	9 / 33	0.24	0.07, 0.80	0.30	0.01, 6.31
Gurage	8 / 18	0.24	0.03, 1.80	1.63	0.09, 31.25
Somali	9 / 10	5.02	0.57, 44.31	0.26	0.01, 7.84
Other	13 / 21	0.63	0.15, 2.60	1.01	0.06, 16.95
		p = 0.55		p = 1.00	
**Alcohol use**					
Never	51 / 79	1.00	Reference	1.00	Reference
Ever	22 / 54	0.45	0.21, 0.96	2.30	0.32, 16.53
		p = 0.04		p = 0.41	
**Staple food**					
Enjera	40 / 93	1.00	Reference	1.00	Reference
Corn bread	9 / 14	1.87	0.67, 5.24	0.95	0.04, 21.35
Kocho	17/16	4.13	1.41, 12.15	7.64	0.73, 79.73
Other	7 / 9	2.01	0.66, 6.18	0.24	0.01, 4.77
Unknown	1 / 1				
		p = 0.03		p = 0.97	
**Porridge**					
No	21 / 47	1.00	Reference	1.00	Reference
Yes	52 / 85	1.46	0.74, 2.88	2.13	0.51, 8.83
Unknown	0 / 1				
		p = 0.94		p = 0.57	
**Green vegetables**					
Daily	27 / 52	1.00	Reference	1.00	Reference
Weekly	11 / 62	0.24	0.08, 0.67	0.22	0.03, 1.49
Less than weekly	21 / 15	3.57	1.31, 9.71	12.68	1.99, 80.96
Not at all	13 / 4	12.47	2.91, 53.39	400	12.00, 13345
Unknown	1 / 0				
		p = 0.00		p = 0.00	
**Saltiness**					
Not salty	20 / 104	1.00	Reference	1.00	Reference
Salty	34 / 24	7.97	3.29, 19.28	7.79	1.21, 50.31
Very salty	19 / 2	1.4 X 10+8	0.00, -	3.9Xe+09	0.00, -
Unknown	0 / 3				
		p = 0.00		p = 0.02	
**Smokiness in home**					
No smoke, very little	12 / 31	1.00	Reference	1.00	Reference
Some smoke	18 / 47	1.23	0.49, 3.09	1.39	0.17, 11.07
A lot of smoke	43 / 55	2.89	1.12, 7.45	1.83	0.28, 11.83
		p = 0.01		p = 0.51	

^a^ Cases of esophageal cancer and gastro-esophageal junction pooled. Inpatient and visitor-type control pooled

^b^ Conditional logistic regression results shown

Education level and religion were strongly associated with risk of EC (Table 3). More educated people had a lower risk of EC compared with illiterate people, even when the level of education did not reach complete schooling. Christians had a lower risk of EC compared with Muslims. Accounting for differences in tobacco use, alcohol use, and consumption of green vegetables did not affect the statistically significant association of education level and religion with risk of EC (Table 3). Thus, education level and religion were associated with EC independently of these specific lifestyle/dietary factors.

## Discussion

Our pilot case–control study showed no excess risk of EC in association with ever use of qat. Cases and controls were generally similar in all qat-related exposures, including duration of use, frequency of use, number of sessions per day, and retention time in the mouth of the qat wad. However, a higher proportion of cases (11%) than of controls (2%) retained the qat wad in the mouth overnight, did not hand-clean the qat leaves before chewing (25% vs 5%), and chewed qat stems (9.6% vs 5.2%). Cigarette smoking during qat chewing sessions was similarly common in cases (12 of 26 ever users; 46%) and in controls (15 of 34 ever users; 44%). Interestingly, in a fully adjusted model limited to never tobacco users, the risk of EC was higher in qat users than in never users, although the precision of this estimate was poor. Thus, acknowledging the small sample size and the exploratory nature of this pilot, the finding of an elevated EC risk with qat use among non-tobacco users deserves discussion.

As a result of habitual qat use, frictional and chemical injury have been reported to induce lesions in the buccal mucosa repeatedly exposed to the plant material, but the pathogenesis of these lesions (oral keratotic white lesions) appears not to be linked to the origin of the neoplasm in the few studies identified [20–23]. In vitro assays on reconstructed human oral mucosa exposed to qat extracts have shown that the plant is capable of modifying the mucosa by producing premature differentiation, atypical keratinization, and senescence, effects mediated by chemical action and resembling mucosal changes seen in the oral cavity of chronic qat users [19,24]. Although the damage caused by friction during prolonged retention of fiber residues is restricted to the mouth, the alkaloids, tannins, and other substances released during chewing are also in contact with the mucosa of the rest of the UDT, including the esophagus. As reported in our study, the qat wad can also be retained in the oral cavity overnight, possibly prolonging the duration of the chemical action. Tannins, with their known astringent properties, are present in significant amounts in qat leaves and have been reported to thicken the mucosa in the esophagus and to cause gastritis in chronic qat users [12,25].

The stimulant active compound in qat, cathinone, is a phenylalkylamine alkaloid [2,26], and a partial involvement in the chemical action described earlier is attributed to this alkaloid, while a potential carcinogenic role has not been reported [19]. The potency of this stimulant compound lasts in the leaves for about 48 hours after harvesting, and later degrades to a less psychoactive form, cathine [27]. The preferred leaves to chew are fresh leaves, chewed within 48 hours after harvesting, but less fresh plant material is also chewed and is sold at a lower price, so qat users may be exposed to both forms of the alkaloid. Other compounds in qat that may exercise a chemical action in the UDT mucosa, and which to our knowledge have not yet been studied for their possible pathogenic role in qat chewers, include other alkaloids (e.g., phenylpentenylamines and cathedulins), terpenoids, flavonoids, sterols, glycosides, amino acids, vitamins, and minerals [27]. Although few animal assays and cell-line studies have indicated that qat exposure induces genotoxic effects, including dominant lethal mutations in mice, embryonic toxicity and teratogenic effects in rats, and nuclear anomalies such as micronuclei in buccal cells [18, 19, 28], the plant constituents responsible for the observed genotoxicity have not been identified.

In addition to natural plant constituents, qat leaves can be contaminated with pesticides during cultivation [29]. In qat grown in different parts of Ethiopia, residual pesticides have been detected, varying in concentration and including high levels, including organophosphate and organochlorine insecticides [30,31]. This is of concern because the time between application of pesticides and harvesting of qat can be as short as one week, as reported by Daba and co-authors [30]. As a custom that uses fresh leaves, which are not washed before use, qat chewing may represent an important source of exposure to pesticides, including DDT and diazinon. Both of these active ingredients were detected in some of the qat specimens collected in Ethiopia in 2009 and examined in the study of Daba et al. [30]. Both active ingredients have recently been classified by IARC as probably carcinogenic to humans, with studies on non-Hodgkin lymphoma, leukemia, and cancers of the lung, liver, and testis providing limited evidence for carcinogenicity in humans [32,33]. The widespread and intense use of pesticides, including DDT, by Ethiopian farmers has been described in production systems of different crops and scales [34], underscoring the need to pay attention to the level of pesticide contamination in qat, which is known to induce psychological dependence [27,35] and consequently continued use and exposure. Risk of EC was assessed in association with established risk factors as well.

As expected, we observed elevated EC risk with ever use of tobacco, an approximately 3-fold excess risk compared with never users. Both cigarette smoking and smokeless tobacco use are established causes of EC [36]. African studies linking tobacco use to cancer occurrence are rare, but the study by Pacella-Norman and collaborators [37] in black South Africans reported a more than 3-fold increase in risk of EC in current and former male smokers. We observed a more than 2-fold increased risk of EC in ever alcohol users compared with never alcohol users. Alcohol consumption is an established cause of cancer in the aerodigestive tract, including the esophagus [36]. Alcoholic beverages expose consumers to ethanol and the metabolized by-product acetaldehyde, itself a genotoxic carcinogen. One study from South Africa addressing the risk of EC in relation to alcohol consumption and concentrating on genetic polymorphisms showed risk estimates of similar magnitude to those reported in our pilot study [38]. Sewram et al. [39] reported higher risk estimates than those in our pilot study for men and women consuming more than 53 g of ethanol per day (OR = 4.72; 95% CI, 2.64, 8.41 for men and OR = 5.24; 95% CI, 3.34, 8.23 for women) compared with non-drinkers, in South Africa.

Low consumption of fruits and vegetables, and in particular of green vegetables, was associated with a substantially increased risk of EC in our pilot study. Consistent with our findings, several studies associated high intake of fruits and vegetables with reduced risk of EC or Barrett’s esophagus [40–43]. For example, Swarm et al. in South Africa documented that the risk of EC in people who consumed green leafy vegetables 5–7 times per week was 38–50% lower than in those who consumed them less than once per week. Fruits and vegetables are sources of antioxidants, vitamins, and minerals, which participate in the detoxifying activity of enzymes and in reducing the damage generated by oxidative stress and by inflammation, among other effects [42].

Consumption of salty or very salty food was associated with a substantial excess EC risk in our study population. However, “very salty” can indicate different levels of salt use to the people interviewed in the study, limiting the interpretation of these results, which are based on a subjective appraisal of the exposure intended for evaluation. Scientific evidence indicates an elevated risk of stomach cancer with excess salt intake, with an up to 2-fold increase in risk [44]. Salt induces gastritis in experimental animals and mediates exacerbation of the carcinogenic effects of known gastric risk factors, such as *Helicobacter pylori*, by increasing inflammation [40]. The association of high salt intake with risk of EC is not documented to a similar extent. Indirect evidence shows an increased risk of squamous cell carcinoma of the esophagus (ESCC) with consumption of salty meats [45]. Drinking of salt tea in India has been linked to excess risk of ESCC [46].

In multivariable analyses, adjusting for potential confounders, we found that the risk of EC was higher in less educated people than in more educated people, and in Muslims than in Christians. There may be other factors associated with low socioeconomic status, education level or income, and religion that were not considered in our analyses, or, as discussed below, the association may be a direct effect of the ascertainment of the study population, due to selection bias.

### Limitations

One weakness of this study is the small sample size, because this was a pilot study, which limits the interpretation of our findings because the precision of the risk estimates is low. Another major limitation is the marked difference in education level and religion between cases and controls, an imbalance that might not be entirely mitigated by adjustment during the analysis. Education level and religion can determine exposure to lifestyle factors associated with cancer risk. This has been clearly shown in this pilot study, where reporting of alcohol use, an established carcinogen, was much less common among Muslims. In our study population, the proportions of subjects reporting very low consumption of green vegetables (38% vs 16%) or specifying *kocho* as the staple food (23% vs 10%) were greater among Muslims than among Christians. Qat chewing was also more commonly reported in Muslim enrollees (45%) than in Christians (16%) in our pilot study, and this has been reported in other studies conducted in Ethiopia [8]. Smokiness inside the home, consumption of very salty food, and *kocho* as the staple food were more commonly reported by illiterate participants than by subjects with any level of formal education.

The language used when responding to the questionnaire also differed between cases and controls, and this imbalance could have introduced bias derived from possibly inaccurate translations, which would apply more often to cases than to controls. This difference may signal different socioeconomic status or education level, assuming that more educated subjects tend to speak not only the local language but also the national language, Amharic. In addition, it may hint of a rural/urban gradient in the countryside, where more cases might have come from rural areas and the matched controls from the urban centers in the same or neighboring zones. For any future studies, the questionnaire should be provided in additional languages other than the country’s official language, Amharic, at a minimum in Oromiya.

Having used as a time reference for the reporting of diet one year before the interview, or one year before the endoscopy or diagnosis, does not necessarily guarantee food frequency consumption data that are indicative of the disease-free period preceding the onset of cancer. Subclinical disease might have been present during this time frame, resulting in lower consumption, and not necessarily that low consumption induced disease. About 68% of EC cases who reported consuming green vegetables less than once a week or not at all (*n* = 34) reported eating beans weekly or more often one year before the interview, suggesting ingestion of solids was possible, and low consumption of green vegetables part of their diet. The low reporting of consumption of solids during the specified time frame may, nevertheless, be due to a growing tumor (reverse causation) and a future questionnaire would also need to ask dietary habits at an earlier time.

### Considerations for future studies

People with suspected EC attending the two clinics and the TAH in Addis Ababa represent a highly selective group of cases from the total pool of EC cases in the country, and this may limit the interpretations of findings in this pilot study. However, the associations with tobacco use, alcohol use, and some other results obtained in our study are as expected based on the literature. Nevertheless, a large-scale study would need to include health centers in a high-risk EC area in the country, such as Bale and Arsi zones in the Oromia Region, with a study questionnaire available in the Oromo language. Such a multi-center setting offers a greater possibility of identifying a group of cancer cases that are more representative of the total pool of cancer cases in the population. Our pilot study suggests an increased EC risk in association with one component of the diet typical of Ethiopia, which merits further evaluation in a properly sized study for verification. Exposure to pesticides in qat chewers also deserves scrutiny in light of the known or suspected carcinogenicity of several active ingredients of pesticides. In view of the high prevalence of qat use in Ethiopia and elsewhere, and the observed non-significantly elevated EC risk in never-tobacco users, it is important to further investigate the role of qat chewing in the occurrence of EC in a study with sufficient statistical power, after taking into account tobacco use and other confounding factors. The choice of control group needs detailed planning to obtain a representative group in terms of education level and religion. This is necessary because several of the assumed risk factors for EC are strongly related to education level and religion, but may be responsible for some of the differences in EC risk between groups with different education levels and different religions. Hospital and endoscopy clinic charts from previous years may be reviewed to create a composition of potential control pools that reflect the general population in relation to those demographic variables.

## Conclusions

In conclusion, our pilot study showed that an EC case–control study in Ethiopia is feasible. Although the results overall showed no excess risk, restricting the analysis to never tobacco users suggested an increased risk in ever qat users compared with never users. A relatively low prevalence of established risk factors for EC was seen in the population in Ethiopia and in our study sample. It is evident that additional, unknown risk factors are present locally. Ethiopia is a good setting for studying the possible carcinogenicity of qat by epidemiological studies, given its widespread use in the population as well as the availability of a large proportion of never users, with relatively stable political conditions and safety for conducting fieldwork. For EC, health centers outside the capital need to be included, because only a selective small proportion of patients make it to Addis Ababa, and this group may not be representative of the totality of cases. Ascertainment of controls remains a challenge, to avoid oversampling by geography, social position, religion, or ethnic group, because many of the potential risk factors are associated with those demographic factors. More work needs to be done to inquire how to ascertain incident oral cancers in an epidemiological study in Ethiopia, because the mouth is the first anatomical site affected by qat chewing. Because qat is commonly chewed by men and women in several countries, this geographical distribution underscores the importance of elucidating a possible role of qat chewing in cancer.

## Supporting information

S1 Fig Residential geographical distribution of esophageal and gastro-esophageal junction cancer cases by zone and region, and matched controls, at the time of enrolment in the pilot study in Addis Ababa in 2012–2013. AFAR REGION = A:Zone 1, B:Zone 2, C: Zone 3; AMHARA REGION = D:North Shewa; OROMIYA REGION = E:Arsi, F:Bale, G:Borena, H:East Harerge, I:East Shewa, J:East Wellega, K:Ilubabor, L:Jimma, M:North Shewa, N:South West Shewa, O:West Arsi, P:West Harerge, Q:West Shewa, R:West Wellega; SOMALI REGION = S:Afder, T:Shabelle (Gode), U:Fafan (Jijiga), V:Siti (Shinile); SNNPR (Southern Nations, Nationalities, and Peoples Region) = W:Gedio, X:Gurage, Y:Hadiya, Z:KT, AA:Selti, AB:Sidama. The designations employed and the presentation of the material in this publication do not imply the expression of any opinion whatsoever on the part of the World Health Organization concerning the legal status of any country, territory, city or area or of its authorities, or concerning the delimitation of its frontiers or boundaries. Shapefile (03 Jun 2014) provided by UN-OCHA and developed by CSA (Central Statists Authority) of Ethiopia. Source: IARC.
(TIF)

S1 File English version of the UDT-Lifestyle questionnaire. (PDF)

S2 File Amharic version of the UDT-Lifestyle questionnaire. (PDF)

S1 Table Description of lifestyle characteristics of study participants included in the analysis. (DOCX)

S2 Table Description of additional lifestyle factors, including dietary parameters, of study participants included in the analysis. (DOCX)
