# Supplementary figures and images for: Qat use and esophageal cancer in Ethiopia: A pilot case-control study
Source: PLoS One. 2017 Jun 8;12(6):e0178911. doi: 10.1371/journal.pone.0178911 (PMC5464578; doi:10.1371/journal.pone.0178911)

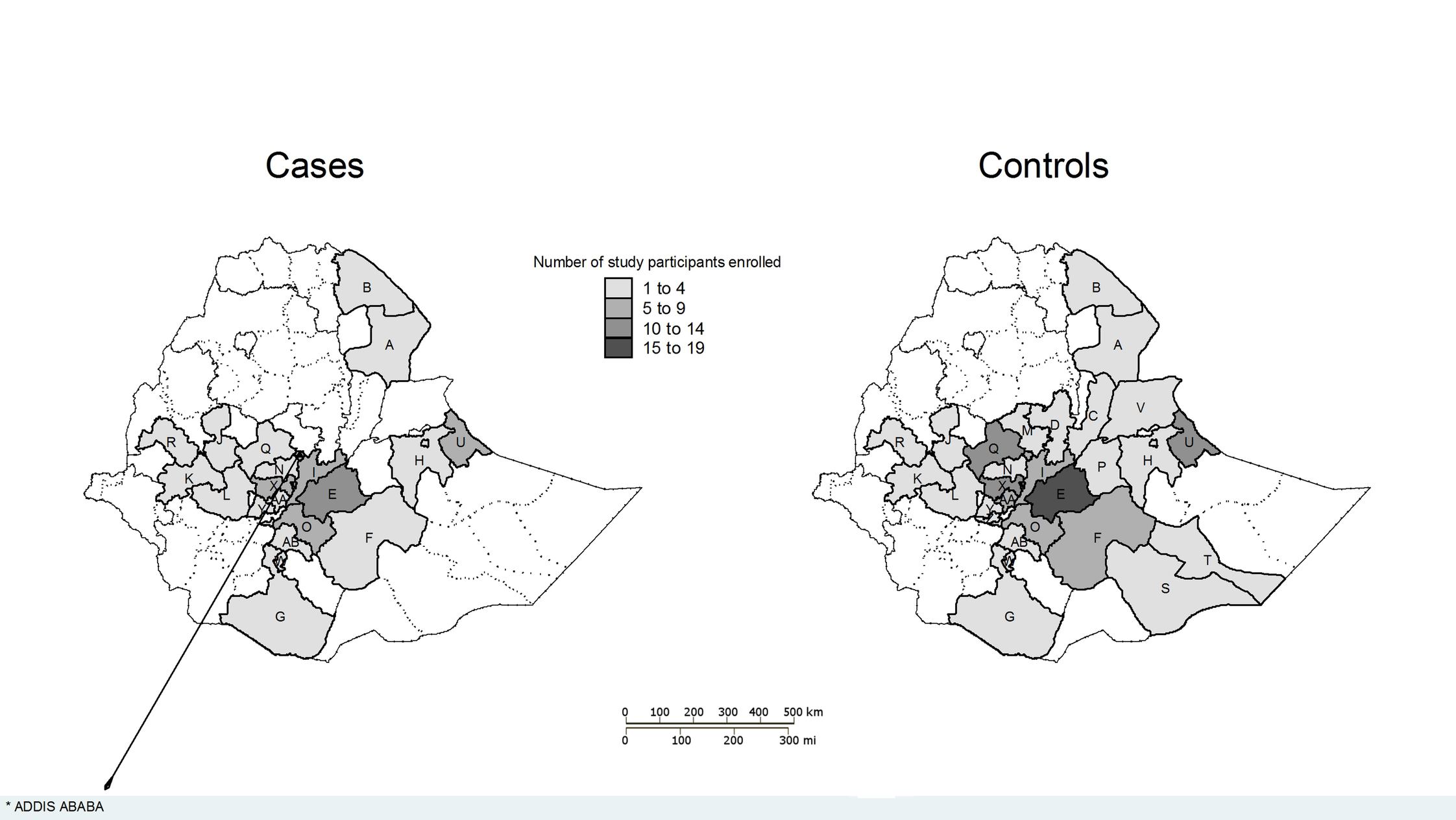

Supplement: S1 Fig — AFAR REGION = A:Zone 1, B:Zone 2, C: Zone 3; AMHARA REGION = D:North Shewa; OROMIYA REGION = E:Arsi, F:Bale, G:Borena, H:East Harerge, I:East Shewa, J:East Wellega, K:Ilubabor, L:Jimma, M:North Shewa, N:South West Shewa, O:West Arsi, P:West Harerge, Q:West Shewa, R:West Wellega; SOMALI REGION = S:Afder, T:Shabelle (Gode), U:Fafan (Jijiga), V:Siti (Shinile); SNNPR (Southern Nations, Nationalities, and Peoples Region) = W:Gedio, X:Gurage, Y:Hadiya, Z:KT, AA:Selti, AB:Sidama. The designations employed and the presentation of the material in this publication do not imply the expression of any opinion whatsoever on the part of the World Health Organization concerning the legal status of any country, territory, city or area or of its authorities, or concerning the delimitation of its frontiers or boundaries. Shapefile (03 Jun 2014) provided by UN-OCHA and developed by CSA (Central Statists Authority) of Ethiopia. Source: IARC. (TIF) [file pone.0178911.s001.tif]
