# Supplementary material for: Qat use and esophageal cancer in Ethiopia: A pilot case-control study
Source: PLoS One. 2017 Jun 8;12(6):e0178911. doi: 10.1371/journal.pone.0178911 (PMC5464578; doi:10.1371/journal.pone.0178911)
Supplement: S1 File — (PDF) [file pone.0178911.s002.pdf]

Lifestyle Factors and Upper Digestive Tract Cancers in Addis Ababa, Ethiopia- A Pilot Case-Control Study

A collaboration among AAU, AHRI, ACS and IARC

Lifestyle questionnaire

Preliminary Part........2
Introduction........3
Part 1. General Information........4
Part 2. Tobacco and Chat use histories........6
Cigarette smoking........6
Water pipe smoking........6
Pipe smoking........7
Chewing Tobacco........7
Chewing Chat........8

Part 3. Alcohol drinking history........12
Binge Drinking........16
Part 4. Drinking tea and coffee history........16
Part 5. Diet history........17
Dietary habits one year ago........18
Part 6. Oral cavity health........19
Part 7. History of various diseases........20
Part 8. Family history of cancer........21
Part 9. Occupational history........22
Part 10. Residential history........24
About the interview........26

Study Number [attach label]

|__| |__||__||__|

Center Subject

Identification numbers should be assigned consecutively within each center.

Center code:

1 – TIKUR ANBESSA SPECIALIZED HOSPITAL

2 – MEXICO HIGHER CLINIC

3 – ADERA HIGHER CLINIC

4 – Dr. NEGA ENT SPECIAL CLINIC

Please fill in 999, 99, or 9, as appropriate, when the subject refuses to answer.
Please fill in 888, 88 or 8, as appropriate, when the subject doesn't know the answer

Preliminary Part

Interviewer: please fill out this part before the interview starts, after consent has been obtained

01001 Status |__| (1) Case (2) Inpatient hospital control (3) Healthy visitor-type control

01002 Endoscopy or laryngoscopy already performed?

|__| (0) no (1) yes (8) don't know (9) refuse to answer

01003 Date of endoscopy or laryngoscopy: dd-mm-yyyy |__|__| - |__|__| - |__|__|__|__|

01004 Clinic or Hospital registration number ________________________________(write number)

01005 Full name of subject: NAME: ____(text)

01006 FATHER'S name: ________________________________(text)

01007 OTHER name: ____ ______________________________(text)

01008 Sex |__| (1) male (2) female

01009 What is your main language ____(text)

ADDRESS:

01010 Sub-city (Kifleketema)__________________;

01011 City______________________________;

01012 Woreda ____________________________ **;**

01013 Kebele____________________________.

01014 Telephone number ____________________________

01015 Interview date: dd-mm-yyyy |__|__| - |__|__| - |__|__|__|__|

01016 Starting Time: |__|__| : |__|__| (12 hr format) Circle if A.M. or P.M.

Pilot case control study of lifestyle factors and upper digestive tract cancer in Addis Ababa, Ethiopia

---

Introduction

Thank you for signing the informed consent to participate in a pilot case control study which is conducted by Addis Ababa University/AAU, Armauer Hansen Research Institute/AHRI, American Cancer Society/ACS and the International Agency for Research on Cancer /IARC in collaboration, to investigate the influence of lifestyle habits and diet on medical conditions and health. We are conducting this research in (this ---------hospital/clinic) and we will interview many patients attending this and other clinics to achieve the objectives of the study.

As we have discussed, the first thing that we will do is to fill in the questionnaire concerning the lifestyle factors which comprises, general information, tobacco smoking history, chat use, alcohol drinking, tea and coffee drinking, last year's dietary habits, medical history, occupational and residential history. I will also measure your stature and take your weight.

I would like to reassure you that the interview will be strictly confidential and that the information disclosed will only be used without any personal name or identifiers.

Any potential benefits of the study for the well-being of the population rely on the accuracy of your answers. Therefore, if you do not understand the meaning of any of the questions, please don't hesitate to ask. At any time you may refuse to continue or to answer specific questions.

Do you have any question/concern?

Yes No

[If the study participant has any concern, the interviewer will give an explanation to clarify or reassure]

If you don't have any question, can we start now?

Part 1. General Information

01017 What is your date of birth?

dd-mm-yyyy |__|__| - |__|__| - |__|__|__|__|

Leave blank if respondent doesn't know the date

01018 What is your age? |__|__|__| years

(888) don't know (999) refuse to answer

01019 What is your marital status |__|

(0) never married (1) married (2) widowed (3) divorced

(4) separated (5) living as married (6) other (8) don't know (9) refuse

01020 What is your ethnicity ____ (text)

01021 What is your religion? |__|

(1) MUSLIM..................... (2) CHRISTIAN (3) OTHER → Specify **01022** : ____________________
(4) NONE .... (9) Refused

01023 What is your education level? [highest level achieved] |__|

(1) illiterate (2) primary school (3) junior middle school (4) senior middle school

(5) college/university and above (8) don't know (9) refused

01024 In which Region do you live? →

Specify: ____________________________________ (text)

01025 In which town do you live? →

Specify: ____(text)

01026 Is your present residence rural or urban? |__|

(1) rural (2) urban (8) don't know (9) refused

How long have you lived in this residence?

01027: Years |__||__|__| or in **01028:** Months |__|__|

01029 In what country were you born→

Specify: ____(text)

01030 In what town or region were you born→

Specify: ____(text)

01031 Height **(cm)** |__|__|__|

(Measured at the time of interview)

01032 Weight **(kg)** |__|__|__|

(Taken at the time of interview)

01033 What was your weight 2 years ago? **(kg)** |__|__|__|

[If you were pregnant at the time, please report your weight for a time around then when you were not pregnant]

01034 What was your weight 5 years ago? **(kg)** |__|__|__|

[If you were pregnant at the time, please report your weight for a time around then when you were not pregnant]

01035 What was your weight at age 20? **(kg)** |__|__|__|

[If you were pregnant at the time, please report your weight for a time around then when you were not pregnant]

Part 2. Tobacco and Chat use histories

Cigarette smoking

02001 |__| Have you ever smoked at least 100 cigarettes in your lifetime?

(0) no (1) yes (8) don't know (9) refused **[If No, go to 02010]**

02002 |__|__|__| On average, how many cigarettes/day do/did you smoke?

02003 |__|__|__| At what age did you start smoking cigarettes?

02004 |__|__|__| How many years have you smoked cigarettes?

(Please make sure to exclude the in-between years of quitting)

02005 |__|__|__| How soon after waking up do you have your first cigarette (in minutes)?

02006 |__| Do you still smoke cigarettes now?

(0) no (1) yes (8) don't know (9) refused

02007 |__| What kind of the cigarettes did/do you smoke?

(1) with filter (2) without filter (3) hand-rolled (9) refuse to answer

Question 2008 only for past smokers [those who answer "no" to Q. 2006];

02008 |__|__|__| How long has it been since you stopped cigarette smoking?

If less than 1 year write number of months **(02009)**_____________(text).

(888) don't know; (999) refuse to answer

Water pipe smoking

02010 |__| Have you smoked water pipe at least once a week for at least 1 year?

(0) no (1) yes (8) don't know (9) refuse to answer **[If No, go to 02022]**

On average,

02011 how many times per week do you smoke a water pipe? **|__|__|__|** times/week

02012 how much quantity of tobacco do/did you use each time? **|__|__|__|** grams/session

02013 |__|__|__| On average, with how many people do/did you share the same pipe during a smoking session?

02014 |__| On average, do/did you smoke it with flavored tobacco, unflavored tobacco, or both?
(1) unflavored (2) flavored (3) both (9) refuse

02015 |__| On average, do/did you mix the water in the tank with other substances?

(0) no (1) yes, Specify **(02016)**_____________ (8) don't know (9) refuse

02017 |__|__|__| How many years have you smoked water pipes?

02018 |__|__|__| At what age did you start smoking water pipes?

02019 |__| Do you still smoke water pipes now?

(0) no (1) yes (8) don't know (9) refuse to answer

Question 02020 only for past water pipe smokers [If "no" to question 02019]

02020 |__|__|__| How long has it been since you stopped water pipe smoking?

If less than 1 year write number of months **(02021)___________ (text);**

(888) don't know; (999) refuse to answer

Pipe smoking

02022 |__| Have you ever smoked at least 50 pipes in your lifetime?

(0) no (1) yes (8) don't know (9) refuse to answer **[If No, go to 02029]**

02023 |__|__|__| On average, how many pipes of tobacco/week do/did you smoke?

02024 |__|__|__| How many years have you smoked a pipe?

02025 |__|__|__| At what age did you start smoking pipes?

02026 |__| Do you still smoke pipes now?

(0) no (1) yes (8) don't know (9) refuse to answer

Question 02027 only for past pipe smokers [If "no" to question 02026];

02027 |__|__|__| How long has it been since you stopped pipe smoking?

If less than 1 year write number of months **(02028)_____________(text);**

(888) don't know; (999) refuse to answer

Chewing Tobacco

02029 |__| Have you chewed tobacco for at least 1 year?

(0) no (1) yes (8) don't know (9) refuse to answer **[If No, go to 02037]**

02030 |__|__| On average, how many times/week did/do you chew tobacco?

02031 |__|__| On average, how many grams of tobacco did/do you chew each time?

02032 |__|__|__| How many years have you chewed tobacco?

02033 |__|__|__| At what age did you start chewing tobacco?

02034 |__| Do you still chew tobacco now?

(0) no (1) yes (8) don't know (9) refuse to answer

Questions 02035 only for past chewers [If "no" to question 02034]

02035 |__|__|__| How long has it been since you stopped chewing tobacco?

If less than 1 year write number of months **(02036)_________(text);** (888) don't know; (999) refuse to answer

Chat use history

Chewing Chat

02037 |__| Have you ever chewed Chat once a week, or more frequently, for at least one year?
(0) no (1) yes (8) don' know (9) refuse to answer **[If No, go to 03001 – page 12]**

02038 |__|__|__| At what age did you start chewing Chat regularly, at least once a week?

02039 |__| Are you chewing Chat regularly these days?

(0) no (1) yes (8) don't know (9) refuse to answer

IF no, **02040 |__|__|__|** years, At what age did you stop chewing Chat?

IF no, **02041** Why did you stop? ____

02042 |__| How do/did you use Chat?

(1) chew fresh tender leaves (2) chew fresh stems
(3) both, chew fresh leaves and stems (4) other

(specify)**(02043)**_____________________________ (9) refuse to answer

What type of Chat leaves do/did you usually consume? Check all that apply.

02044|__| green leaves (0) no (1) yes (8) don't know (9) refuse

02045|__| crimson (dimaa) color leaves (0) no (1) yes (8) don't know (9) refuse

02046|__| dalota (dalacha) (0) no (1) yes (8) don't know (9) refuse

02047|__| hamarcot (0) no (1) yes (8) don't know (9) refuse

02048|__| small size leaves (0) no (1) yes (8) don't know (9) refuse

02049|__| large size leaves (0) no (1) yes (8) don't know (9) refuse

02050|__| other (specify)**(02051)**__________________ (0) no (1) yes (8) don't know (9) refuse

From which of these areas does/did the Chat you chew come from? Check all that apply.

02052|__| Gurage (0) no (1) yes (8) don't know (9) refuse

02053|__| Harer (0) no (1) yes (8) don't know (9) refuse

02054|__| Wendo (0) no (1) yes (8) don't know (9) refuse

02055|__| other (specify)**(02056)**___________________ (0) no (1) yes (8) don't know (9) refuse

02057 |__| On average, how many days per week do/did you use Chat?

Please specify number of days (8) don't know or (9) refuse to answer

02058 |__|__| Typically, how many times do/did you chew on a day (chewing sessions)?
Please enter number of times (88) don't know (99) refuse to answer

How long do/did you chew/ keep the wad of Chat in the mouth during daily session(s)?
If less than 1 hour, enter in minutes, otherwise enter in hours

	**In Minutes**	**Number of hours**
First session	02059 |__|__|<1	02060 |__|__|
Second session	02061 |__|__|<1	02062 |__|__|
Third session	02063 |__|__|<1	02064 |__|__|
Fourth session	02065 |__|__|<1	02066 |__|__|

02067 |__| Do/did you ever leave the wad of Chat in your mouth **overnight**?
(0) no (1) yes (8) don't know (9) refuse to answer

On a typical **chewing session**, on average, how much Chat do/did you use and how much does/did it cost you?

Chat quality, quantity, cost	Fresh leaves, best quality	Fresh leaves, medium quality	Fresh leaves, low quality	Fresh stems	Other
(0) no<br>(1) yes<br>(8) don't know<br>(9) refuse	**02068** |__|	**02073** |__|	**02078** |__|	**02083** |__|	**02088** |__|
Bundles, **number**	**02069**|__|__|	**02074**|__|__|	**02079**|__|__|	**02084**|__|__|	**02089**|__|__|
Bundles, **size**<br>(1) small<br>(2) medium<br>(3) large<br>(8) don't know<br>(9) refuse	**02070**|__|	**02075**|__|	**02080**|__|	**02085**|__|	**02090**|__|
Bundles, **cost** (Birr)	**02071**|__|__|__|	**02076**|__|__|__|	**02081**|__|__|__|	**02086**|__|__|__|	**02091**|__|__|__|
Bundles, **shared** w/ others?<br>(0) no<br>(1) yes<br>(8) don't know<br>(9) refuse	**02072** |__|	**02077** |__|	**02082** |__|	**02087** |__|	**02092** |__|

02093 |_|__|__|__|__| Birr On average, how much do/did you spend on Chat on a chewing day?

On a 24-hour (day) interval, on average, when do/did you usually chew Chat?

Time	Week days (0) no (1) yes (8) don't know (9) refuse to answer	Weekend (0) no (1) yes (8) don't know (9) refuse to answer
Morning	02094 |__|	02095|__|
Afternoon	02096 |__|	02097|__|
Evening	02098 |__|	02099|__|
All day	02100 |__|	02101|__|

02102 |__| Do/did you wash the Chat leaves /stem before chewing?

(0) no (1) yes, always (2) yes, usually (3) yes, sometimes (4) yes, rarely (8) don't know (9) refuse

02103 |__| Do/did you hand-clean the Chat leaves /stem before chewing?

(0) no (1) yes, always (2) yes, usually (3) yes, sometimes (4) yes, rarely (8) don't know (9) refuse

02104 |__| Where in the mouth do/did you usually retain the wad of Chat munch after chewing?

(1) Cheek right side (2) Cheek left side (3) Both sides (4) Elsewhere, **(02105)**_____________
(text)

02106 |__| Do/did you swallow the **Chat juice**?

(0) no (1) yes, always (2) yes, usually (3) yes, sometimes (4) yes, rarely (8) don't know (9) refuse

02107 |__| Do/did you swallow the **Chat residues**?

(0) no (1) yes, always (2) yes, usually (3) yes, sometimes (4) yes, rarely (8) don't know (9) refuse

Do/did you drink any beverage during a Chat chewing session?

	(0) no (1) yes (8) don't know (9) refuse to answer	Number of glasses or cups
Water	02108 |__|	02109|__|__|
Tea	02110 |__|	02111|__|__|
Coffee/qisher	02112 |__|	02113|__|__|
Soft drinks	02114 |__|	02115|__|__|
Other **(02116)** Specify________________	02117 |__|	02118|__|__|

02119 |__| Do/did you smoke cigarettes during Chat chewing sessions?

(0) no (1) yes, always (2) yes, usually (3) yes, sometimes (4) yes, rarely (8) don't know (9) refuse

02120 |__| Do/did you smoke water pipe during Chat chewing sessions?

(0) no (1) yes, always (2) yes, usually (3) yes, sometimes (4) yes, rarely (8) don't know (9) refuse

02121 |__| Do/did you chew tobacco during Chat chewing sessions?

(0) no (1) yes, always (2) yes, usually (3) yes, sometimes (4) yes, rarely (8) don't know (9) refuse

02122 |__| Do/did you have recurrent oral health problems?

(0) no (1) yes, ulcer type (2) yes, cavities (3) yes, other

02123 (most common side in mouth affected)____________________________

02124 Specify other problem ____

Part 3. Alcohol drinking history

03001 |__| Have you ever drank alcohol (*Tela, Tej, Areke,* beer, hard liquor) at least once per week for more than six months?

(0) no (1) yes (8) don't know (9) refuse to answer **[If No, go to 04001]**

03002|__|__| On average, how many drinks (bottles of beer/glasses of tela, wine or liquor) per week do you drink? **(*Please show picture of alcohol beverages)**

03003|__|__| At what age did you begin drinking alcohol?

03004|__| Do you still drink alcohol now?

(0) no (1) yes (8) don't know (9) refuse to answer

Only for past drinkers [If "no" to question03004]

03005 |__|__| How many years has it been since you stopped drinking?

Now I would like to ask you about the types of alcoholic beverages you drank in your lifetime. *For each of the beverages shown in the table below please ask questions A to F, if applicable*

a) Did/do you ever drink ___?

b) From what age did you start drinking ____?

c) How many drinks per week did/do you drink of _____ at that time?

d) Please specify the size of the drink for your ____ consumption.

[interviewer – please show picture of alcoholic beverages]

e) **[Only for 'other' drinks]** What was the % alcohol for this type of alcoholic beverage?

f) Did you quit drinking, or did you increase or decrease the amount of drinking?

If yes, how old were you when you quit or changed? **(please, repeat from question B to F if changed pattern)**

<table>
<tr><th></th><th>A</th><th>B</th><th>C</th><th>D</th><th>E</th><th>F</th></tr>
<tr><th>Alcoholic Beverage Type</th><th>(0) no
(1)yes
(8) don't know
(9) refuse</th><th>From age</th><th>Drinks per week*</th><th>Size of each drink in ml</th><th>% alcohol in each drink (write %)</th><th>To age</th></tr>
<tr><td>Beer</td><td>03006 |__|</td><td>03007 |__|__|</td><td>03008 |__|__|</td><td>03009 |__|__|__|</td><td>03010 ________</td><td>03011 |__|__|__|</td></tr>
<tr><td></td><td></td><td>03012 |__|__|</td><td>03013 |__|__|</td><td>03014 |__|__|__|</td><td>03015 ____</td><td>03016 |__|__|__|</td></tr>
<tr><td></td><td></td><td>03017 |__|__|</td><td>03018 |__|__|</td><td>03019 |__|__|__|</td><td>03020 ____</td><td>03021 |__|__|__|</td></tr>
<tr><td></td><td></td><td>03022 |__|__|</td><td>03023 |__|__|</td><td>03024 |__|__|__|</td><td>03025 ____</td><td>03026 |__|__|__|</td></tr>
<tr><td></td><td></td><td>03027 |__|__|</td><td>03028 |__|__|</td><td>03029 |__|__|__|</td><td>03030 ____</td><td>03031 |__|__|__|</td></tr>
<tr><td>Tela</td><td>03032 |__|</td><td>03033 |__|__|</td><td>03034 |__|__|</td><td>03035 |__|__|__|</td><td>03036____</td><td>03037 |__|__|__|</td></tr>
<tr><td></td><td></td><td>03038 |__|__|</td><td>03039 |__|__|</td><td>03040 |__|__|__|</td><td>03041____</td><td>03042 |__|__|__|</td></tr>
<tr><td></td><td></td><td>030043 |__|__|</td><td>03044 |__|__|</td><td>03045 |__|__|__|</td><td>03046____</td><td>03047 |__|__|__|</td></tr>
<tr><td></td><td></td><td>03048 |__|__|</td><td>03049 |__|__|</td><td>03050 |__|__|__|</td><td>03051____</td><td>03052 |__|__|__|</td></tr>
<tr><td></td><td></td><td>03053 |__|__|</td><td>03054 |__|__|</td><td>03055 |__|__|__|</td><td>03056____</td><td>03057 |__|__|__|</td></tr>
<tr><td>Wine</td><td>03058 |__|</td><td>03059 |__|__|</td><td>03060 |__|__|</td><td>03061 |__|__|__|</td><td>03062____</td><td>03063 |__|__|__|</td></tr>
<tr><td></td><td></td><td>03064 |__|__|</td><td>03065 |__|__|</td><td>03066 |__|__|__|</td><td>03067____</td><td>03068 |__|__|__|</td></tr>
<tr><td></td><td></td><td>03069 |__|__|</td><td>03070 |__|__|</td><td>03071 |__|__|__|</td><td>03072____</td><td>03073 |__|__|__|</td></tr>
<tr><td></td><td></td><td>03074 |__|__|</td><td>03075 |__|__|</td><td>03076 |__|__|__|</td><td>03077____</td><td>03078 |__|__|__|</td></tr>
<tr><td></td><td></td><td>03079 |__|__|</td><td>03080 |__|__|</td><td>03081 |__|__|__|</td><td>03082____</td><td>03083 |__|__|__|</td></tr>
<tr><td>Tej</td><td>03084 |__|</td><td>03085 |__|__|</td><td>03086 |__|__|</td><td>03087 |__|__|__|</td><td>03088____</td><td>03089 |__|__|__|</td></tr>
<tr><td></td><td></td><td>03090 |__|__|</td><td>03091 |__|__|</td><td>03092 |__|__|__|</td><td>03093____</td><td>03094 |__|__|__|</td></tr>
<tr><td></td><td></td><td>03095 |__|__|</td><td>03096 |__|__|</td><td>03097 |__|__|__|</td><td>03098____</td><td>03099 |__|__|__|</td></tr>
<tr><td></td><td></td><td>03100 |__|__|</td><td>03101 |__|__|</td><td>03102 |__|__|__|</td><td>03103____</td><td>03104 |__|__|__|</td></tr>
<tr><td></td><td></td><td>03105 |__|__|</td><td>03106 |__|__|</td><td>03107 |__|__|__|</td><td>03108____</td><td>03109 |__|__|__|</td></tr>
<tr><td>Hard Liquor</td><td>03110 |__|</td><td>03111 |__|__|</td><td>03112 |__|__|</td><td>03113 |__|__|__|</td><td>03114____</td><td>03115 |__|__|__|</td></tr>
<tr><td></td><td></td><td>03116 |__|__|</td><td>03117 |__|__|</td><td>03118 |__|__|__|</td><td>03119____</td><td>03120 |__|__|__|</td></tr>
<tr><td></td><td></td><td>03121 |__|__|</td><td>03122 |__|__|</td><td>03123 |__|__|__|</td><td>03124____</td><td>03125 |__|__|__|</td></tr>
</table>

	A	B	C	D	E	F
Alcoholic Beverage Type	(0) no<br>(1)yes<br>(8) don't know<br>(9) refuse	From age	Drinks per week*	Size of each drink in ml	% alcohol in each drink (write %)	To age
		03126 |__|__|	03127 |__|__|	03128 |__|__|__|	03129____	03130 |__|__|__|
		03131 |__|__|	03132 |__|__|	03133 |__|__|__|	03134____	03135 |__|__|__|
Areke	03136 |__|	03137 |__|__|	03138 |__|__|	03139 |__|__|__|	03140____	03141 |__|__|__|
		03142 |__|__|	03143 |__|__|	03144 |__|__|__|	03145____	03146 |__|__|__|
		03147 |__|__|	03148 |__|__|	03149 |__|__|__|	03150____	03151 |__|__|__|
		03152 |__|__|	03153 |__|__|	03154 |__|__|__|	03155____	03156 |__|__|__|
		03157 |__|__|	03158 |__|__|	03159 |__|__|__|	03160____	03161
Other (specify)	03162 |__|					
**Text ________________ 03163**		03164 |__|__|	03165 |__|__|	03166 |__|__|__|	031767____	03168 |__|__|__|
**Text ________________ 03169**		03170 |__|__|	03171 |__|__|	03172 |__|__|__|	03173____	03174 |__|__|__|
**Text ________________ 03175**		03176 |__|__|	03177 |__|__|	03178 |__|__|__|	03179____	03180 |__|__|__|
**Text ________________ 03181**		03182 |__|__|	03183 |__|__|	03184 |__|__|__|	03185____	03186 |__|__|__|
**Text ________________ 03187**		03188 |__|__|	03189 |__|__|	03190 |__|__|__|	03191____	03192 |__|__|__|

Alcoholic Beverages

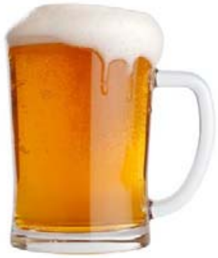	BEER/LAGER/CIDER<br>- ½ pint (285 ml)<br>- 1 pint (570 ml)<br>- 1 small bottle/1 small can (330 ml)<br>- 1 large bottle/1 large can (500 ml)
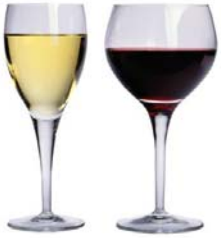	WINE (include Babycham, Champagne)<br>- 1 small glass (125 ml)<br>- 1 large glass (250 ml)<br>- 1 bottle = 6 small glasses (750 ml)
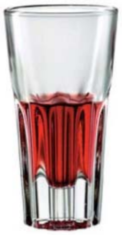	APERITIFS, PRE DINNER DRINKS (vermouth, martini, cinzano, dubonnet, pastis, etc.)<br>- 1 single measure (25 ml)<br>- 1 double measure (50 ml)<br>- 1 bottle = 28 single measures (700 ml)
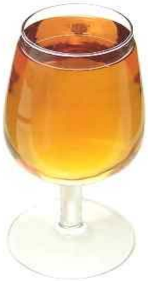	FORTIFIED WINE (sherry, port, other fortified or tonic wine)<br>- 1 small glass (50 ml)
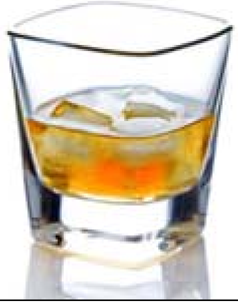	SPIRITS/LIQUORS<br>- 1 single measure (25 ml)<br>- 1 larger measure (35 ml)<br>- 1 double measure (50 ml)<br>- 1 bottle = 28 single measures (700 ml)

Binge Drinking

03193 |__| Have you ever drank 5 drinks or more in a single occasion?
(0) no (1) yes (8) don't know (9) refuse to answer **[If No, go to 04001]**

03194 |__|__| If yes, on average how may drinks per occasion?

03195 |__|__| How many times per year?

03196 |__|__|__| At what age did you start this drinking pattern?

03197 |__|__| How many years have you been drinking in this pattern?

Part 4. Drinking tea and coffee history

Tea

04001|__| Have you ever drank at least one cup of tea a week, for six months or more?
(0) no (1) yes (8) don't know (9) refuse to answer **[If No, go to 04013]**

04002|__|__|__| At what age did you start drinking tea?

04003|__|__|__| How many years have you drank tea?

04004|__|__|__| How many cups of tea do you drink per week, on average?

04005 |__|__|__|__| What is the volume (ml) of the cup you use to drink tea on average?
(8888) don't know; (9999) refuse to answer

Do you drink:

04006 |__| Black tea
(0) no (1) yes (8) don't know (9) refuse to answer

04007 |__| Green tea
(0) no (1) yes (8) don't know (9) refuse to answer

04008 |__| Flower tea
(0) no (1) yes (8) don't know (9) refuse to answer

04009 |__| Herbal infusions
(0) no (1) yes (8) don't know (9) refuse to answer

04010 |__| Other
(0) no (1) yes (8) don't know (9) refuse to answer
04011 Specify ________________________

04012 |__| Do/did you usually drink hot or cold tea?

(1) cold tea (2) warm tea (3) hot tea (4) very hot tea (8) don't know (9) refuse to answer

Coffee

04013 |__| Have you ever drank at least one cup of coffee a week, for six months or more?

(0) no (1) yes (8) don't know (9) refuse to answer **[If No, go to 05001]**

04014|__|__|__| years At what age did you start drinking coffee?

04015|__|__|__| How many years have you drank coffee?

04016|__| Do/did you roast your coffee at home? (0) no (1) yes;

04017 If yes, how many times per week do/did you roast coffee at home? **|__|__| __|**

04018|__| Do/did you usually brew coffee at home?

(0) no (1) yes (8) don't know (9) refuse to answer

04019|__| Do/did you usually drink coffee at bars/coffee house/restaurant

(0) no (1) yes (8) don't know (9) refuse to answer

04020|__| Do/did you usually drink coffee at other places? (specify)

(0) no (1) yes (8) don't know (9) refuse to answer

04021 If yes, specify ___________________________________

04022 |__| Do/did you usually drink coffee with:

(1) sugar only (2) milk/cream only (3) salt (4) sugar and milk (5) salt and milk (8) don' know (9) refuse

04023|__| On average, do/did you drink strong or light coffee?

(1) strong coffee (2) moderate coffee (3) light coffee (4) not sure (8) don't know (9) refuse

04024 |__|__|__| On average, how many cups of coffee do/did you drink per week?

04025|__|__|__|__| What is/was the volume (ml) of the cup you use to drink coffee in, on average?

(8888) don't know; (9999) refuse to answer

Part 5. Diet history

Please recall your diet history **one year ago** when responding to the following questions.

(For cases: 1 year before the cancer diagnosis)

05001 |__| What was the staple food you used to eat at your home:

(1) Enjera (2) Corn bread (3) Kotcho (4) other (specify)**(05002)**_____________ (8) don't know (9) refused

05003 |__| The food you ate in general was

(1) very salty (2) salty (3) not very salty (4) not salty (8) don't know (9) refused

05004 |__| Did you eat porridge (genfo) at least once a month?

(0) no (1) yes (8) don't know (9) refuse to answer **[If no, go to Question 05007]**

05005 |__|__| If yes, how many times per month did you eat porridge? **05006 |__|** How did you eat the porridge?

(1) very hot (2) hot (3) cold (8) don't know (9) refuse to answer

05007 |__| Did you use refrigerators to preserve food?

(0) no (1) yes (8) don't know (9) refuse to answer **[If no, go to item 05009]**

05008 |__|__| If so, how many years have you stored food in the refrigerator over your lifetime?

05009 |__|__| How many people were usually eating together in your home?

<u>Dietary habits one year ago</u>

One year ago, how frequently did you consume food from the following categories?

Category	One year ago, the frequency of consumption (Choose one frequency for each category)									
	3 meals everyday	2 meals everyday	1 meal everyday	5-6 times per week	3-4 times per week	1-2 times per week	Less than once a week	Not at all	Don't Know	Refuse
05010 Meat (beef, lamb, goat)	|__|1	|__|2	|__|3	|__|4	|__|5	|__|6	|__|7	|__|8	|__|9	|__|10
05011 Chicken	|__|1	|__|2	|__|3	|__|4	|__|5	|__|6	|__|7	|__|8	|__|9	|__|10
05012 Fish	|__|1	|__|2	|__|3	|__|4	|__|5	|__|6	|__|7	|__|8	|__|9	|__|10
05013 Milk or other dairy products	|__|1	|__|2	|__|3	|__|4	|__|5	|__|6	|__|7	|__|8	|__|9	|__|10
05014 Eggs (from chicken or duck)	|__|1	|__|2	|__|3	|__|4	|__|5	|__|6	|__|7	|__|8	|__|9	|__|10
05015 Green leafy vegetables	|__|1	|__|2	|__|3	|__|4	|__|5	|__|6	|__|7	|__|8	|__|9	|__|10
05016 Other vegetables	|__|1	|__|2	|__|3	|__|4	|__|5	|__|6	|__|7	|__|8	|__|9	|__|10

05017 Fruits	|__|1	|__|2	|__|3	|__|4	|__|5	|__|6	|__|7	|__|8	|__|9	|__|10
05018 Beans or bean products	|__|1	|__|2	|__|3	|__|4	|__|5	|__|6	|__|7	|__|8	|__|9	|__|10
05019 Preserved/ fermented food	|__|1	|__|2	|__|3	|__|4	|__|5	|__|6	|__|7	|__|8	|__|9	|__|10

Part 6. Oral cavity health

06001 |__| How often do you clean your teeth?

(0) never (1) < once a week (2) 1 to 2 times a week (3) every other day

(4) once a day (5) 2 times a day (6) 3 times a day (7) > 3 times a day

(8) don' know (9) refused

06002 |__|__| How many teeth do you currently have? (normally between 28-32)

[Interviewer: please help them count if they do not know and consent to counting]

06003 |__| Do you wear dentures?

(0) no (1) yes (8) don' know (9) refuse to answer **[If no, please go to 06005]**

06004 |__|__|__| At what age did you start wearing dentures?

06005 |__| How often do you visit a dentist?

(0) never (1) once every ≥5 years (2) once every 2-4 years

(3) once a year (4) more than once a year (8) don't know (9) refused

Part 7. History of various diseases

Now, I would like to ask about your health in the past

(A) Has a doctor ever told you that you had any of the following conditions?
(B) If yes, how old were you when you first had this problem?
(C) Did you take medication for it?
(D) What was the medication?
(E) Did you have surgery for it?

(A) Past disease<br>(0) no<br>(1) yes<br>(8) don't know<br>(9) refuse	(B) Age in years<br>888 if don't know	(C) Medication<br>(0) no<br>(1) yes<br>(8) don't know<br>(9) refuse	(D) specify medication (text) or if applicable, don't know	(E) Surgery<br>(0) no<br>(1) yes<br>(8) don't know<br>(9) refuse
07001 |__| High blood pressure	[07002] |__|__|__|	[07003] |__|	[07004]	[07005] |__|
07006 |__| Diabetes<br>(not including gestational diabetes)	[07007] |__|__|__|	[07008] |__|	[07009]	[07010] |__|
07011 |__| Tuberculosis	[07012] |__|__|__|	[07013] |__|	[07014]	[07015] |__|
07016 |__| Repetitive dental ulcer	[07017] |__|__|__|	[07018] |__|	[07019]	[07020] |__|
07021 |__| Oral leukoplakia	[07022] |__|__|__|	[07023] |__|	[07024]	[07025] |__|
07026 |__| Oral submucous fibrosis (OSF)	[07027] |__|__|__|	[07028] |__|	[07029]	[07030] |__|
07031 |__| Depression	[07032] |__|__|__|	[07033] |__|	[07034]	[07035] |__|
07036 |__| Asthma	[07037] |__|__|__|	[07038] |__|	[07039]	[07040] |__|
07041 |__| Any allergy	[07042] |__|__|__|	[07043] |__|	[07044]	[07045] |__|

Part 8. Family history of cancer

Please tell me the number of brothers, sisters, sons and daughters that you have (exclude step-brothers and step-sisters):

08001 Brothers |__|__| **08002** Sisters |__|__| **08003** Sons |__|__| **08004** Daughters |__|__|

08005 Have any of your first degree relatives (those specified below) had a diagnosis of cancer? |__|
(0) no (1) yes (8) don't know (9) refused **[If no, go to 09001]**

Please list all your first degree relatives who ever had a diagnosis of cancer:
(1) Father (2) Mother (3) Brother (4) Sister (5) Son (6) Daughter

Relative (code 1-6)	[08006] |__|	[08013] |__|	[08020] |__|	[08027] |__|	[08034] |__|	[08041] |__|	[08048] |__|
First Cancer site (write text)	[08007]	[08014]	[08021]	[08028]	[08035]	[08042]	[08049]
Age at diagnosis	[08008] |__|__|	[08015] |__|__|	[08022] |__|__|	[08029] |__|__|	[08036] |__|__|	[08043] |__|__|	[08050] |__|__|
Cancer site (ICD-9)	**[Study Coordinator– please code after the interview]**						
	[08009] |__|__|__|	[08016] |__|__|__|	[08023] |__|__|__|	[08030] |__|__|__|	[08037] |__|__|__|	[08044] |__|__|__|	[08051] |__|__|__|
Second Cancer site (text)	[08010]	[08017]	[08024]	[08031]	[08038]	[08045]	[08052]
Age at diagnosis	[08011] |__|__|	[08018] |__|__|	[08025] |__|__|	[08032] |__|__|	[08039] |__|__|	[08046] |__|__|	[08053] |__|__|
Cancer site (ICD-9)	**[Study Coordinator – please code after the interview]**						
	[08012] |__|__|__|	[08019] |__|__|__|	[08026] |__|__|__|	[08033] |__|__|__|	[08040] |__|__|__|	[08047] |__|__|__|	[08054] |__|__|__|
*Please attach additional sheets if needed.							

Part 9. Occupational history

09001 **|__|__|__|** At what age did you start working full time [more than 20 hours a week]?

09002 **|__|** Are you currently working?

(0) no (1) yes (8) don't know (9) refused **[If yes, please go to 10001]**

09003 **|__|__|__|** If you no longer work, at what age did you stop working full time [more than 20 hours a week]?

09004 **|__|__|__|** How many years did you work in total?

Please tell me about any jobs you held for at least one year starting from the first job to your most recent job.

(A) At what age did you start this job?
(B) At what age did you stop this job?
(C) What was your job title?
(D) What was the type of industry for this job?

(A) From (age)	(B) To (age)	(C) Occupation (fill in text & use codes)	(D) Industry (fill in text & use codes)	Codes
09005|__|__|	09006|__|__|	09007|__|__| __________<br>09008	09009|__|__| __________<br>09010	**Occupation coding: (ISCO 1968)**<br>1. Professional, technical and related workers<br>2. Administrative and managerial workers<br>3. Clerical and related workers<br>4. Sales workers<br>5. Service workers<br>6. Agricultural (farming), animal husbandry and forestry workers, fishermen and hunters<br>7. Production and related workers<br>8. Transport equipment operators and labourers<br>9. Armed forces<br>10. Other<br>88. Don't know<br>99. Refuse to answer<br><br>**Industry coding: (ISIC)**<br>1. Agriculture (farming), Hunting, Forestry and Fishing<br>2. Mining and Quarrying<br>3. Manufacturing<br>4. Electricity, Gas and Water<br>5. Construction<br>6. Wholesale and retail trade<br>7. [illegible]
09011|__|__|	09012|__|__|	09013|__|__| __________<br>09014	09015|__|__| __________<br>09016	
09017|__|__|	09018|__|__|	09019|__|__| __________<br>09020	09021|__|__| __________<br>09022	
09023|__|__|	09024|__|__|	09025|__|__| __________<br>09026	09027|__|__| __________<br>09028	
09029|__|__|	09030|__|__|	09031|__|__| __________<br>09032	09033|__|__| __________<br>09034	

(A) From (age)	(B) To (age)	(C) Occupation (fill in text & use codes)	(D) Industry (fill in text & use codes)	Codes
09035|__|__|	09036|__|__|	09037|__|__| ______________<br>09038	09039|__|__| ______________<br>09040	
09041|__|__|	09042|__|__|	09043|__|__| ______________<br>09044	09045|__|__| ______________<br>09046	
09047|__|__|	09048|__|__|	09049|__|__| ______________<br>09050	09051|__|__| ______________<br>09052	
09053|__|__|	09054|__|__|	09055|__|__| ______________<br>09056	09057|__|__| ______________<br>09058	
09059|__|__|	09060|__|__|	09061|__|__| ______________<br>09062	09063|__|__| ______________<br>09064	

*Please attach additional sheets if needed.

Part 10. Residential history

Please tell me where you have lived for at least one year, starting with your birthplace and finishing with your present address (please include your birthplace and present residence regardless the length of time you live there).

A) In which city, country did you live?
B) From what age, did you start to live in that location?
C) At what age, did you move to a different residence?
D) Is the city size considered urban or rural?
E) What cooking method was mainly used in the residence?
F) How smoky was it inside the house?
G) Is the kitchen inside your home?

E) Cooking methods
1=Natural gas
2=Electricity
4=Wood stove
5=Microwave oven*
6=Electromagnetic oven*
7=Charcoal
8=Propane
9=Seldom cook at home
10=Other
88=Don't know
99=Refused

F) Smokiness in home
0=no smoke,
1=very little smoke
2=some smoke
3=a lot of smoke
8=don't know
9=refused

<table>
<tr><th>(A) City and region (or country if living outside Ethiopia)
[please enter text]</th><th>(B)
From
(age)</th><th>(C)
To
(age)</th><th>(D)
City Size
(0) urban
(1) rural)
(8) don't know
(9) refused</th><th>(E) Cooking method</th><th>(F)
Smokiness in home</th><th>(G)
Is kitchen inside home
(0) no
(1) yes
(8) don't know
(9) refused</th></tr>
<tr><td>[10001-10002]</td><td>[10003]
|__|__|</td><td>[10004]
|__|__|</td><td>[10005]
|__|</td><td>[10006]
|__||__|</td><td>[10007]
|__|</td><td>[10008]
|__|</td></tr>
<tr><td>[10009-10010]</td><td>[10011]
|__|__|</td><td>[10012]
|__|__|</td><td>[10013]
|__|</td><td>[10014]
|__||__|</td><td>[10015]
|__|</td><td>[10016]
|__|</td></tr>
<tr><td>[10017-10018]</td><td>[10019]
|__|__|</td><td>[10020]
|__|__|</td><td>[10021]
|__|</td><td>[10022]
|__||__|</td><td>[10023]
|__|</td><td>[10024]
|__|</td></tr>
<tr><td>[10025-10026]</td><td>[10027]
|__|__|</td><td>[10028]
|__|__|</td><td>[10029]
|__|</td><td>[10030]
|__|__|</td><td>[10031]
|__|</td><td>[10032]
|__|</td></tr>
</table>

(A) City and region (or country if living outside Ethiopia) [please enter text]	(B) From (age)	(C) To (age)	(D) City Size (0) urban (1) rural) (8) don't know (9) refused	(E) Cooking method	(F) Smokiness in home	(G) Is kitchen inside home (0) no (1) yes (8) don't know (9) refused
[10033-10034]	[10035] |__|__|	[10036] |__|__|	[10037] |__|	[10038] |__|__|	[10039] |__|	[10040] |__|
[10041-10042]	[10043] |__|__|	[10044] |__|__|	[10045] |__|	[10046] |__|__|	[10047] |__|	[10048] |__|
[10049-10050]	[10051] |__|__|	[10052] |__|__|	[10053] |__|	[10054] |__||__|	[10055] |__|	[10056] |__|
[10057-10058]	[10059] |__|__|	[10060] |__|__|	[10061] |__|	[10062] |__|__|	[10063] |__|	[10064] |__|
[10065-10066]	[10067] |__|__|	[10068] |__|__|	[10069] |__|	[10070] |__|__|	[10071] |__|	[10072] |__|
[10073-10074]	[10075] |__|__|	[10076] |__|__|	[10077] |__|	[10078] |__|__||	[10079] |__|	[10180] |__|

*Please attach additional sheets if needed.

END of the questionnaire: Thank you very much for the time you have given us!

About the interview [Please complete immediately after the study participant is thanked]

[11001] Name of interviewer: ....

[11002] Date of interview completed (dd/mm/yyyy) |__|__|/|__|__|/|__|__|__|__|

[11003]Time of finishing the interview: |__|__| : |__|__|

(12 hr format) Circle if A.M. or P.M.

[11004] How long did the interview take? |__|__| hours: |__|__| min

[11005] **|__|** Was there anyone present (other than you and the participant) during any portion of the interview?

(0) No (1) Yes (8) don't know (9) refuse to answer

[11006] **|__|__|** If yes, who?

(1) mother (2) father (3) sister (4) brother (5) daughter
(6) son (7) grandparents (8) spouse (9) friend
(10) hospital staff (11) other

[11007] **|__|** Was the interview conducted in a language other than Amharic?

(0) No (1) Yes (8) don't know (9) refuse to answer

[11008] If yes, in which language? ____________________________ (text)

[11009] Who did the translation? ______________________________(text)

[11010] **|__|** Quality of cooperation: 0=poor, 1=fair, 2=good

[11011] Any additional comments about this interview [text]:

____

____

____

____
