# Supplementary material for: Qat use and esophageal cancer in Ethiopia: A pilot case-control study
Source: PLoS One. 2017 Jun 8;12(6):e0178911. doi: 10.1371/journal.pone.0178911 (PMC5464578; doi:10.1371/journal.pone.0178911)
Supplement: S2 File — (PDF) [file pone.0178911.s003.pdf]

በኢትዮጵያ ውስጥ የላይኛው ስርዓተ-እንሽርሽሪት አካል ካንሰር ካለባቸውና ከሌለባቸው ሰዎች ላይ ስለ ኑሮ ሁኔታቸው የሚደረግ የሙከራ ጥናት

በአዲስ አበባ ዩኒቨርሲቲ ፣ በአርማዎር ሃንሰን የምርምር ተቋም፣ በአሜሪካ የካንሰር ማህበረሰብ እና በአለም አቀፍ የካንሰር ምርምር ድርጅት ትብብር የሚካሔድ ጥናት

የአኗኗር ሁኔታ ቃለመጠይቅ

ማ ው ጫ

የቃለ መጠየቁ መግቢያ-- 2

መግቢያ -- 4

ክፍል 1. አጠቃላይ መረጃ-- 5

ክፍል 2. ትንባሆ የማጨስ ታሪክን በተመለከተ-- 6

ሲጋራ ማጨስን በተመለከተ -- 6
ሺሻ መጠቀምን በተመለከተ -- 7
ፒፓ ማጨስን በተመለከተ-- 8
ትንባሆን ማኘክን በተመለከ-- 9
ጫት መጠቀምን በተመለከተ -- 9

ክፍል 3. አልኮል የመጠጣት ታሪክ -- 15

የአልኮል መጠጥ በብዛት የሚጠጣበትን ክፍለ ጊዜ በተመለከተ-- 20

ክፍል 4. የሻይና ቡና የመጠጣት ታሪክ-- 20

ክፍል 5. የአመጋገብ ሁኔታን በተመለከተ -- 22

ከአንድ አመት በፊት የነበረ የምግብ አመጋገብ -- 23

ክፍል 6. የአፍ ጤንነትን በተመለከተ-- 24

ክፍል 7. የተለያ በሽታዎች ታሪክን በተመለከተ-- 25

ክፍል 8. የቤተሰብ የካንሰር ታሪክ -- 26

ክፍል 9፡ የስራ ታሪክ-- 27

ክፍል 10፡ የመኖሪያ አካባቢ ታሪክ -- 30

ስለ ቃለ መጠይቅ አድራጊው/ዋ-- 33

የጥናቱ ተሳታፊ መለያ ቁጥር(መለያ ባር ኮድ ይለጠፍበታል)

|__||__| |__||__||__|__|__|

ማእከል   የጥናቱ ተሳታፊ መለያ ቁጥር

በእያንድንዱ የጥናቱ ማእከል የመለያ ቁጥሩ በሚሰጥበት ጊዜ ተከታታይ መሆን አለበት፡፡

<u>የማእከሉ ቁጥር</u>

01- ጥቁር አንበሳ ስፔሻላይዝድ ሆስፒታል

02- ሜክሲኮ ከፍተኛ ክሊኒክ

03-አደራ ከፍተኛ ክሊኒክ

04- ደ/ር ነጋ ከአንገት በላይ ህክምና ልዩ ክሊኒክ

እባክዎ የጥናቱ ተሳታፊ ጥያቄን ለመመለስ ፈቃደኛ በማይሆኑበት ወቅት 999፣ 99 ወይም 9 ብለው እንደ አግባብነቱ ይፃፉ፡፡

የጥናቱ ተሳታፊ የጥያቄውን መልስ ካላወቁት 888፣ 88 ወይም 8 ብለው እንደ አግባብነቱ ይፃፉ፡፡

<u>የቃለ መጠየቁ መግቢያ</u>

እባክዎ ይህንን የመጠይቅ ክፍል የጥናቱ ተሳታፊ ለመሳትፍ የስምምነት ቅፅ ከፈረሙ በኃላ <u>ከቃለ መጠይቁ በፊት ይሙሉት!</u>

01001 የተሳትፎው አይነት |__|

1) የላይኛው ስርዓተ-እንሽርሽሪት አካል ካንሰር ያለባቸው ተሳታፊዎች
2) ካንሰር የሌለባቸውና ለሌላ ህክምና ተኝተው የሚታከሙ ተሳታፊዎች
3) ካንሰር የሌለባቸው ጤናማ ተሳታፊዎች

01002 ኢንዶስኮፒ (ላርይንጎስኮፒ) ተካሔዷል? |__| (0) አይደለም (1) አይደለም

01003 ኢንዶስኮፒ (ላርይንጎስኮፒ) የተካሔደበት ቀን ቀን /ወር/ዓ.ም

|__|__| - |__|__| - |__|__|__|__|

01004 የሆስፒታል/የክሊኒክ መለያ ቁጥር ________________________________(በቁጥር)
01005 የጥናቱ ተሳታፊ ሙሉ ስም፡ ስም፡ ________________________________(በፅሁፍ)

01006 የአባት ስም፡_____________________________(በፅሁፍ)

01007 ሌላ ስም፡________________________________(በፅሁፍ)

01008 ፆታ|__| (1)=ወንድ (2)= ሴት

01009 የሚጠቀሙበት ዋናው ቋንቋ ___________________________________

አድራሻ

01010 ክፍለ ከተማ፡________________________

01011 ከተማ፡_________________________

01012 ወረዳ፡ ___________________________

01013 ቀበሌ፡___________________________

01014 ስልክ ቁጥር፡ _________________________________

01015 የቃለ-መጠይቁ ቀን፡- ቀን /ወር/ዓ.ም |__|__| - |__|__| - |__|__|__|__|

01016 ቃለ-መጠይቁ የተጀመረበት ሠዓት |__|__|: |__|__| (በ 12፡00 ሰዓት አቆጣጠር)

በአዲስ አበባ ፣ኢትዮጵያ ውስጥ የላይኛው ስርዓተ-እንሽርሽሪት አካል ካንሰር ካለባቸውና ከሌለባቸው ሰዎች ላይ ስለ ኑሮ ሁኔታቸው የሚደረግ የሙከራ ጥናት

መግቢያ

በአዲስ አበባ ዩኒቨርሲቲ፣ በአርማዎር ሃንሰን የምርምር ተቋም፣ በአሜሪካ የካንሰር ማህበረሰብ እና በአለም አቀፍ የካንሰር ምርምር ድርጅት ትብብር የአኗኗር፣ የአመጋገብ፣ የህክምና ሁኔታ እና በበሽታና በጤና ላይ ያላቸውን ተጽእኖ በአዲስ አበባ ከተማ ለማጥናት በሚደረገው የሙከራ ጥናት ላይ ለመሳተፍ ፈቃደኝነትዎትን በፊርማዎ ስላረጋገጡልን እናመሰግናለን፡፡ የዚህን ጥናት ዓላማ ለማሳከት ሲባል በጥቁር አንበሳ ስፔሻሻላይዝድ ሆስፒታል፣ በሜክሲኮ ከፍተኛ ክሊኒክ፣ በአደራ ከፍተኛ ክሊኒክ እና በዶ/ር ነጋ ከአንገት በላይ ህክምና ልዩ ክሊኒክ የሚገኙ የተለያዩ ህመምተኞችን ቃለ መጠይቅ የምናደርግ ይሆናል፡፡

ቀደም ሲል እንደተነጋገርነው በመጀመሪያ ቃለ መጠይቁን የምናደርግ ሲሆን ጥያቄዎቹም አጠቃላይ መረጃ፣ ትንባሆ የማጨስ ታሪክን፣ ጫት የመጠቀም ታሪክን፣ አልኮል የመጠጣት ታሪክን፣ ሻይ እና ቡና የመጠጣት ታሪክ፣ የባለፈው ዓመት የአመጋገብ ሁኔታ፣ የህክምና ታሪክ፣ የስራና የመኖሪያ አካባቢ ታሪክን የሚያጠቃልሉ ይሆናሉ፡፡ ከዚህም በተጨማሪ ቁመትዎን እና ክቢደትዎን እለካለሁ፡፡

ደግሜ ላረጋግጥልዎ የምፈልገው ነገር ቢኖር ከቃለ መጠይቁ ላይ የሚገኙ ማናቸውም መረጃዎች በሙሉ ምስጥራዊነታቸው የሚጠበቅ ሲሆን በፅሁፍ ላይ የሚወጣው መረጃ ደግሞ በስምዎ ወይም ደግሞ በእርስዎ መገለጫ እነደማይሆን ነው፡፡

የዚህ ጥናት ለሰው ልጆች የሚሰጠው ጥቅም እርስዎ በሚሰጡት ትክክለኛ ምላሽ ላይ የተመሰረተ ነው፡፡ ስለዚህ ግልፅ ያልሆነ ጥያቄ ካለ ለመጠየቅ እንዳያመናቱ እና በማንኛውም ጊዜ ቃለ መጠይቁን ማ ቋረጥ ወይንም መመለስ የማይፈልጉት ጥያቄ ካላ ምላሽ አለመስጠት ይችላሉ፡፡

ግልፅ ያልሆነ ነገር ወይንም ጥያቄ አለዎት፡፡

|__|አዎ |__|አይደለም

[የጥናቱ ተሳታፊ ጥያቄ ካነሱ፤ ቃለ መጠይቅ አድራጊው ስለ ጥያቄዎቸ ምላሽ ይሰጣል ወይም ትሰጣለች በተጨማሪም ተሳታፊ እርግጠኛ እንዲሆን ይደረጋል፡፡]

ጥያቄ ከሌለዎት አሁን መጀመር እንችላለን?

ክፍል 1. አጠቃላይ መረጃ

01017 የትውልድ ዘመን፡- ቀን /ወር/ዓ.ም |__|__| - |__|__| - |__|__|__|__|

የጥናቱ ተሳታፊ የትዉልድ ዘመናቸውን የማያውቁት ከሆነ፡ ክፍት ይተውት፡፡

01018 እድሜ |__|__|__| (በዓመት)

(888) አላውቅም (999) ለመመለስ ፈቃደኛ አይደሉም

01019 የጋብቻ ሁኔታ |__|

(0) ያላገባ (1) ያገባ (2) ባሏ/ ሚስቱ በሞት የተለያት/የተለየችው

(3) የተፋታ/የተፋታች (4) ባለ ትዳር ሆነው ተለያይው የሚኖሩ (5) እንደተጋቡ አብረው የሚኖሩ (6) ሌላ (8) አላውቅም (9) ፈቃደኛ አይደሉም

01020 ብሔር __________________________________ (በፅሁፍ)

01021 ሃይማኖት |__|

(1) ሙስሊም (2) ክርስቲያን (3) ሌላ 01022፡ _________________________

(4) ምንም (9) ፈቃደኛ አይደሉም

01023 የደረሱበት ከፍተኛ የትምህርት ደረጃ |__|

(1) ያልተማረ/ች (2) አንደኛ ደረጃን ያጠናቀቀ/ች (3) የመለስተኛ ደረጃን ያጠናቀቀ/ች

(4) የሁለተኛ ደረጃን ያጠናቀቀ/ች (5) ኮሌጅ/ዩኒቨርሲቲ/ከዛ በላይ ያጠናቀቀ/ች

(8) አላውቅም (9) ለመመለስ ፈቃደኛ አይደሉም

01024 የሚኖሩት በየትኛው ክልል ውስጥ ነው?_____________________ (በፅሁፍ)

01025 በአሁኑ ሰዓት የመኖሪያ ከተማ ? ________________________ (በፅሁፍ)

01026 የሚኖሩበት ቦታ ገጠር ወይንስ ከተማ ነው? |__|

(1) ገጠር (2) ከተማ (8)አላውቅም (9) ለመመለስ ፈቃደኛ አይደሉም

አሁን በሚኖሩበት አካባቢ ለስንት ጊዜ ኖረዋል?

01027፡ አመታት |__||__|__| 01028፡ ወራት |__|__|

01029 የተወለዱበት ሀገር ______________________ (በፅሁፍ)

01030 የተወለዱበት ከተማ (ክልል) ________________________ (በፅሁፍ)

01031 ቁመት (በሴ.ሜ) |__|__|__|

(በቃለ መጠይቁ ጊዜ የሚለካ)

01032 ክብደት (በኪ.ግ) |__|__|__|

(በቃለ መጠይቁ ጊዜ የሚለካ)

01033 ከሁለት ዓመት በፊት የነበረዎን ክብደት (**በኪሎ ግራም**) |__|__|__|

(በወቅቱ ነብሰ-ጡር ከነበሩ ፤ እባክዎ ነብሰ-ጡር ባልነበሩበት የነበረ ክብደትዎን ይንገሩን!)

01034 ከአምስት ዓመት በፊት የነበረዎን ክብደት (**በኪሎ ግራም**) |__|__|__|

(በወቅቱ ነብሰ-ጡር ከነበሩ ፤ እባክዎ ነብሰ-ጡር ባልነበሩበት የነበረ ክብደትዎን ይንገሩን!)

01035 በ20 ዓመት እድሜዎ ክብደትዎ (**በኪሎ ግራም**) ስንት ነበር? |__|__|__|

(በወቅቱ ነብሰ-ጡር ከነበሩ፤ እባክዎ ነብሰ-ጡር ባልነበሩበት የነበረ ክብደትዎን ይንገሩን!)

ክፍል 2. ትንባሆ የማጨስ ታሪክን በተመለከተ

ሲጋራ ማጨስን በተመለከተ

02001 |__| በህይወትዎ ቢያንስ 100 ሲጋራዎችን አጭሰዋል?

(0) አይደለም (1) አዎ (8) አላውቅም (9) ለመመለስ ፈቃደኛ አይደሉም

(የተሳታፊው ምላሹ" አይደለም"" ከሆነ ወደ ጥያቄ ቁጥር 02010 ይሒዱ !)

02002 |__|__|__| በአማካኝ በአንድ ቀን ውስጥ ምን ያህል ሲጋራ አጭሰው ነበር/ እያጨሱ ይገኛሉ?

02003 |__|__|__| ሲጋራ ማጨስ የጀመሩት በስንት አመትዎ ነው?

02004 |__|__|__| ለምን ያህል አመት ሲጋራን አጭሰዋል?

(በመካከል ያላጨሱበት ጊዜ ካለ እባክዎ ያንን ጊዜ በመቀነስ ይንገሩን!)

02005 |__|__|__| ከእንቅልፍዎ ከተነሱ በኃላ የመጀመሪያ ሲጋራዎን በስንት ደቂቃ ውስጥ ያጨሳሉ?

02006 |__| በአሁኑ ሰዓት ሲጋራ እያጨሱ ይገኛሉ?

(0) አይደለም (1) አዎ (8) አላውቅም (9) ለመመለስ ፈቃደኛ አይደሉም

02007 |__| የሚያጨሱት ሲጋራ ምን አይነት ነው?

(1) ማጣሪያ ያለው (2) ማጣሪያ የሌለው (3) በእጅ የሚጠቀለል (9)ለመመለስ ፈቃደኛ አይደሉም

ጥያቄ ቁጥር 02008 የቀድሞ ሲጋራ አጫሾችን ይመለከታል፡፡(ማለትም ለጥያቄ ቁጥር 02006 "አይደለም " ብለው መልስ ለሰጡ)

02008 |__|__|__| ሲጋራ ማጨስ ካቆሙ ምን ያህል አመት ሆነዎት?

ከአንድ ዓመት ያነሰ ከሆነ የወሮችን ብዛት(02009)________________(በፅሁፍ)

(888) አላውቀውም (999))ለመመለ ፈቃደኛ አይደሉም

<u>ሺሻ መጠቀምን በተመለከተ</u>

02010 |__| ቢያንስ ለአንድ ዓመት ያህል በሳምንት ቢያንስ አንድ ጊዜ ሺሻን

አጭሰዋል? (0) አይደለም (1) አዎ (8) አላውቀውም (9) ለመመለስ ፈቃደኛ አይደሉም **(የተሳታፊው/ዋ ምላሽ "አይደለም" ከሆነ እባክዎ ወደ ጥያቄ ቁጥር 02022 ይሒዱ!)**

በአማካኝ

02011 በአንድ ሳምንት ውስጥ ምን ያህል ጊዜ እያጨሱ ይገኛሉ? |__|__|__| ጊዜ/በሳምንት

02012 በአንድ ሳምንት ውስጥ ምን ያህል ግራም ትንባሆ እያጨሱ ይገኛሉ? |__|__|__| ግራም/በአንድ የማጨሻ ክፍለ ጊዜ

02013 |__|__|__| በአማካኝ በአንድ የሺሻ ማጨሻ ክፍለ ጊዜ አንድ ሺሻ መጨሻን

ከስንት ሰው ጋር በጋራ ይጠቀማሉ?

02014 |__| በአማካኝ የሚያጨሱት የሺሻ አይነት (1) ጥሩ ማእዛ የሌላቸውን (2) ጥሩ ማእዛ ያላቸው (3) ሁለቱንም አያነት (9) ለመመለስ ፈቃደኛ አይደሉም

02015 |__| በአማካኝ ሺሻ በሚያጨሱበት ወቅት በእቃው ውስጥ ውሃውን ከሌላ ነገር ጋር አቀላቅለው ያውቃሉ?

(0) አያደለም
(1) አዎ፣ የሚያቀላቅሉበትን ነገር ይግለፁልን (02016)________________
(8) አላውቅም (9) ለመመለስ ፈቃደኛ አይደሉም

02017 |__|__|__| ለምን ያህል ዓመት ሺሻን አጭሰዋል?

02018 |__|__|__| ሺሻን ማጨስ የጀመሩት በስንት ዓመትዎ ነው?

02019 |__| በአሁኑ ጊዜ ሺሻን ያጨሳሉ?

(0) አይደለም (1) አዎ (8) አላውቅም (9) ለመመለስ ፈቃደኛ አይደሉም

ጥያቄ ቁጥር 02020 ለቀድሞ ሺሻ ተጠቃሚዎች ብቻ የሚጠየቅ ነው፡፡ (ለጥያቄ ቁጥር 02019 ምላሻቸው አይደለም ለነበረ ተሳፊዎች)

02020 |__|__|__| ሺሻ ማጨስ ካቆሙ ስንት ዓመት ሆነዎት? ሺሻን ማጨስ ያቆሙት ከ1 ዓመት በታች ከሆነ፤ በወር ይቀመጥ (02021) __________(**በፅሁፍ**)

(888) አላውቅም (999))ለመመለ ፈቃደኛ አይደሉም

<u>ፒፓ ማጨስን በተመለከተ</u>

02022 |__| በህይወትዎ ቢያንስ 50 ፒፓዎችን አጭሰዋል?

(0) አይደለም (1) አዎ (8) አላውቅም (9) ለመመለስ ፈቃደኛ አይደሉም

(ምላሹ አይደለም ከሆነ ወደ ጥያቄ ቁጥር 02029 ይሒዱ)

02023 |__|__|__| በአማካይ በአንድ ሳምንት ውስጥ ምን ያህል ፒፓ አጭሰው ነበር/ እያጨሱ ይገኛሉ?

02024 |__|__|__| ለምን ያህል አመት ፒፓን አጭሰዋል?

02025 |__|__|__| ፒፓ ማጨስ የጀመሩት በስንት አመትዎ ነው?

02026 |__| በአሁኑ ሰዓት ፒፓ እያጨሱ ይገኛሉ?

(0) አይደለም (1) አዎ (8) አላውቅም (9) ለመመለስ ፈቃደኛ አይደሉም

ጥያቄ ቁጥር 02027 የቀድሞ ፒፓ አጫሾችን የሚመለከት ነው፡፡(ለጥያቄ ቁጥር 02026 ምላሻቸው አይደለም ለነበረ ተሳፊዎች)

02027 |__|__|__| ፒፓ ማጨስ ካቆሙ ስንት ዓመት ሆነዎት?

ፒፓን ማጨስ ያቆሙት ከ1 ዓመት በታች ከሆነ፤በወር ይቀመጥ
(02028)______________________ (**በፅሁፍ**)

(888) አላውቀውም (999) ለመመለ ፈቃደኛ አይደሉም

ትንባሆን ማኘክን በተመለከ

02029 |__| ቢያንስ ለአንድ ዓመት ያህል ትንባሆን አኝከዋል?

(0) አይደለም (1) አዎ (8) አላውቅም (9) ለመመለስ ፈቃደኛ አይደሉም

(የተሳታፊው/ዋ ምላሽ "አይደለም" ከሆነ ወደ ጥያቄ ቁጥር 02037 ይሒዱ)

02030 |__|__| በአመካኝ በአንድ ሳምንት ውስጥ ምን ያህል ጊዜ ትንባሆ አኝከዋል/

እያኙኩ ይገኛሉ?

02031 |__|__| በአማካኝ ትንባሆ በሚጠቀሙበት ወቅት ምን ያህል ግራም ትንባሆ

አኝከዋል/እያኙኩ ይገኛሉ?

02032 |__|__|__| ለምን ያህል ዓመት ትንባሆን አኝከዋል?

02033 |__|__|__| ትንባሆ ማኘክ የጀመሩት በስንት ዓመትዎ ነው?

02034 |__| በአሁኑ ጊዜ ትንባሆ እያኙኩ ይገኛሉ?

(0) አይደለም (1) አዎ (8) አላውቅም (9) ለመመለስ ፈቃደኛ አይደሉም

ጥያቄ ቁጥር 02035 ለቀድሞ ትንባሆን በማኘክ ለተጠቀሙት ብቻ የሚጠየቅ ነው፡፡(ለጥያቄ ቁጥር 02034 ምላሻቸው አይደለም ለነበረ ተሳፊዎች)

02035 |__|__|__| ትንባሆ ማኘክ ካቆሙ ስንት ዓመት ሆነዎት?

ትምባሆ ማኘክን ያቆሙት ከ1 ዓመት በታች ከሆነ፤በወር ይቀመጥ
(02036)____________________ (**በፅሁፍ**)

(888) አላውቀውም (999) ለመመለስ ፈቃደኛ አይደሉም

ጫት መጠቀምን በተመለከተ

02037 |__| በሳምንት አንድ ጊዜ ወይንም በጣም በተደጋጋሚ ቢያንስ ለአንድ አመት

ጫት ተጠቅመዋል?

(0) አይደለም (1) አዎ (8) አላውቅም (9) ለመመለስ ፈቃደኛ አይደሉም

(የተሳታፊወ ምላሽ አየደለም ከሆነ ወደ ጥያቄ ቁጥር 03001 ማለትም ወደ ገፅ 15 ይሂዱ!)

02038 |__|__|__| ቢያንስ በሳምንት አንድ ጊዜ ጫት መጠቀም ማለትም

በተከታታይነት የጀመሩት በስንት ዓመትዎ ነበር?

02039 |__| በአሁኑ ሰአት ጫት በተደጋጋሚ ይጠቀማሉ?

(0) አይደለም (1) አዎ (8) አላውቅም (9) ለመመለስ ፈቃደኛ አይደሉም

መልስዎ አይደለም ከሆነ፤ 02040 |__|__|__| ዓመት/ ጫት መጠቀም ሲያቆሙ ስንት ዓመትዎ ነበር? መልስዎ አይደለም ከሆነ፤ 02041 ጫት መጠቀምዎን ለምን አቆሙ?____

02042 |__| ጫትን እንዴት ተጠቅመውበታል ወይንም እየተጠቀሙበት ነው?

(1) ለጋ ቅጠሉን በማላመጥ (2) ለጋ ገረባውን በማላመጥ (3) ለጋ ቅጠሉንና ገረባውን በማላመጥ (4) ሌላ (02043)_______________(ይግለፁልን) (9) ለመመለስ ፈቃደኛ አይደሉም

ቀድሞ ወይንም በአሁን ጊዜ ምን አይነት ጫት ተጠቅመዋል ወይንም እየተጠቀሙ ነው? <u>እባክዎ የሚጠቀሙትን አይነት በሙሉ ምልክት ያድርጉበት!</u>

02044	|__|	አረጊዴ ቅጠል	0) አይደለም	(1) አዎ	(8) አላውቅም	(9) ፈቃደኛ አይደሉም
02045	|__|	ደማቅ ቀይ(ዲማ) ቅጠሎችን	0) አይደለም	(1) አዎ	(8) አላውቅም	(9) ፈቃደኛ አይደሉም
02046	|__|	ዳሎት (ዳልቻ)	0) አይደለም	(1) አዎ	(8) አላውቅም	(9) ፈቃደኛ አይደሉም
02047	|__|	ሃማርኮት	0) አይደለም	(1) አዎ	(8) አላውቅም	(9) ፈቃደኛ አይደሉም
02048	|__|	ትንንሽ ቅጠሎችን	0) አይደለም	(1) አዎ	(8) አላውቅም	(9) ፈቃደኛ አይደሉም
02049	|__|	ትልልቅ ቅጠሎችን	0) አይደለም	(1) አዎ	(8) አላውቅም	(9) ፈቃደኛ አይደሉም
02050	|__|	ሌላ ካለ (ይግለፅሉን)	0) አይደለም	(1) አዎ	(8) አላውቅም	(9) ፈቃደኛ አይደሉም
		(02051)_____________				

የተጠቀሙበት ወይንም እየተጠቀሙበት ያለው ጫት ከየት አካባቢ የሚመጣ ነው?

02052	|__|	ጉራጌ	0) አይደለም	(1) አዎ	(8) አላውቅም	(9) ፈቃደኛ አይደሉም
02053	|__|	ሃረር	0) አይደለም	(1) አዎ	(8) አላውቅም	(9) ፈቃደኛ አይደሉም
02054	|__|	ወንዶ	0) አይደለም	(1) አዎ	(8) አላውቅም	(9) ፈቃደኛ አይደሉም
02055	|__|	ሌላ ካለ (ይግለፅሉን)	0) አይደለም	(1) አዎ	(8) አላውቅም	(9) ፈቃደኛ አይደሉም
		(02056)_________				

02057 |__| በአማካይ <u>በአንድ ሳምንት ውስጥ ምን ያክል ቀን</u> ጫት ተጠቅመዋል ወይንም እየተጠቀሙ ነው? እባክዎ የቀኖችን ቁጥር ይግለፁልን፡፡

(8) አላውቅም (9) ለመመለስ ፈቃደኛ አይደሉም

02058 |__|__| በአንድ ቀን ውስጥ ጫትን የተጠቀሙበት ወይንም የሚጠቀሙበት ጊዜ ምን ያክል ነው? (በምን ያክል ጊዜ አዳዲስ ጫት ወደ አፍዎ ይጨምራሉ?) እባክዎ የሚጠቀሙበትን ጊዜ ቁጥር ይግለፁልን፡፡ (88) አላውቀውም (99) ለመመለስ ፈቃደኛ አይደሉም

የተላመጠውን ጫት በየቀኑ የጫት መጠቀሚያ ክፍለ ጊዜ ውስጥ ለምን ያህል ሰዓት አፍዎ ውስጥ ያቆዩታል? አፍዎ ውስጥ የሚቆዩት ከአንድ ሰዓት ያነሰ ከሆነ ደቂቃውን ይንገሩን፣ አለበለዚያ ግን በሰዓት ይንገሩን፡፡

ክፍለ ጊዜ	ደቂቃ	ሰዓት
የመጀመሪያው ክፍለ ጊዜ	02059 |__|__|<1	02060 |__|__|
ሁለተኛው ክፍለ ጊዜ	02061 |__|__|<1	02062 |__|__|
ሶስተኛው ክፍለ ጊዜ	02063 |__|__|<1	02064 |__|__|
አራተኛው ክፍለ ጊዜ	02065 |__|__|<1	02066 |__|__|

02067 |__| የተላመጠውን ጫት አፍዎ ውስጥ **አሳድረውት** ያውቃሉ?

(0) አይደለም (1) አዎ (8) አላውቅም (9) ለመመለስ ፈቃደኛ አይደሉም

በአንድ ጫት የመጠቀሚያ ክፍለ ጊዜ በአማካይ ምን ያክል ጫት ተጠቅመዋል ወይንም ይጠቀማሉ? ዋጋውስ?

የጫት ጥራት፣ ብዛትና ዋጋ	አዲስ የተቆረጠ ቅጠል፣ በጣም ጥራት ያለው	አዲስ የተቆረጠ ቅጠል፣ መካከለኛ ጥራት	አዲስ የተቆረጠ ቅጠል፣ ዝቅተኛ ጥራት	አዲስ የተቆረጠ ገረባ	ሌላ
(0) አይደለም<br>(1) አዎ<br>(8) አላውቅም<br>(9) ፈቃደኛ አይደሉም	02068 |__|	02073 |__|	02078 |__|	02083 |__|	02088<br>|__|
የእስሩ ቁጥሩ	02069|__|__|	02074|__|__|	02079 |__|__|	02084|__|__|	02089|__|__|
የእስሩ መጠን<br>(1) አነስተኛ<br>(2) መካከለኛ<br>(3) ትልቅ<br>(8) አላውቅም<br>(9) ፈቃደኛ አይደሉም	02070 |__|	02075 |__|	02080 |__|	02085|__|	02090 |__|
የእስሩ ዋጋ(ብር)	02071|__|__|__|	02076|__|__|__|	02081|__|__|__|	02086|__|__|__|	02091|__|__|__|
እስሩን ሌላ ሰው ጋር ተጋርተዋል?<br>(0) አይደለም<br>(1) አዎ<br>(8) አላውቅም<br>(9) ፈቃደኛ አይደሉም	02072 |__|	02077 |__|	02082|__|	02087 |__|	02092 |__|

02093 |__|__|__|__| ብር፣ ጫት በሚጠቀሙበት ቀን በአንድ ቀን ውስጥ ለጫት በአማካኝ ምን ያህል ገንዘብ ሲያወጡ ነበር ወይንም እያወጡ ይገኛሉ?

በአንድ ቀን(በ24 ሰዓት ውስጥ) በየትኛው ሰዓት ውስጥ ጫት ይጠቀማሉ?

ጊዜ	ከ ሰኞ እስከ አርብ<br>(0) አይደለም (1) አዎ	ቅዳሜና እሁድ<br>(0) አይደለም (1) አዎ
ጠዋት	02094 |__|	02095 |__|__|
ከሰዓት በኃላ	02096 |__|	02097 |__|__|
ምሽት	02098 |__|	02099 |__|__|
ሙሉ ቀን	02100 |__|	02101 |__|__|

02102 |__| የጫቱን ቅጠል ወይንም ገረባውን ከመጠቀምዎ በፊት ያጥቡታል?

(0) አይደለም (1) አዎ፡ ሁልጊዜ (2) አዎ፡ በተደጋጋሚ

(3) አዎ፡ አልፎ አልፎ (4) አዎ፡ በጣም ጥቂት ጊዜ

(8) አላውቅም (9) ለመመለስ ፈቃደኛ አይደሉም

02103 |__| የጫቱን ቅጠል ወይን ገረባ ከመጠቀምዎ በፊት በእጅዎን አሽተው ያፀዱታል?

(0) አይደለም (1) አዎ፡ ሁልጊዜ (2) አዎ፡ በተደጋጋሚ

(3) አዎ፡ አልፎ አልፎ (4) አዎ፡ በጣም ጥቂት ጊዜ

(8) አላውቅም (9) ለመመለስ ፈቃደኛ አይደሉም

02104 |__| ጫት ከተጠቀሙ በኃላ የታለመጠውን ጫት በየትኛው የአፍዎ ክፍል ያስቀምጡታል? (1) በቀኝ በኩል (2) በግራ በኩል (3) በሁለቱም በኩል

(4) በሌላ ቦታ (02105) ___________________________________ (ይግለፁልን)

02106 |__| **የጫቱን ፈሳሽ** ይውጡታል/ውጠውት ያውቃሉ?

(0) አይደለም (1) አዎ፡ ሁልጊዜ (2) አዎ፡ በተደጋጋሚ

(3) አዎ፡ አልፎ አልፎ (4) አዎ፡ በጣም ጥቂት ጊዜ

(8) አላውቅም (9) ለመመለስ ፈቃደኛ አይደሉም

02107 |__| ጫቱ ከ**ተላመጠ በኃላ ቀሪውን ክፍል** ይውጡታል/ውጠውት ያውቃሉ?

(0) አይደለም (1) አዎ፡ ሁልጊዜ (2) አዎ፡ በተደጋጋሚ

(3) አዎ፡ አልፎ አልፎ (4) አዎ፡ በጣም ጥቂት ጊዜ

(8) አላውቅም (9) ለመመለስ ፈቃደኛ አይደሉም

ጫት በሚጠቀሙበት ወቅት የሚጠጡ ነገሮችን ወስደዋል ወይንም ይወስዳሉ?

	(0) አይደለም (1) አዎ<br>(8) አላውቅም<br>(9) ለመመለስ ፈቃደኛ አይደሉም	የብርጭቆ የስኒ ብዛት በቁጥር
ውሃ	02108 |__|	02109 |__|__|
ሻይ	02110 |__|	02111 |__|__|
ቡና/ቀሽር	02112 |__|	02113 |__|__|
ለስላሳ መጠጥ	02114 |__|	02115 |__|__|
ሌላ(02116)_________________	02117 |__|	02118 |__|__|

02119 |__| ጫት በሚጠቀሙበት ጊዜ ሲጋራ ያጨሳሉ/ ያጨሱ ነበር?

(0) አይደለም (1) አዎ፡ ሁልጊዜ (2) አዎ፡ በተደጋጋሚ

(3) አዎ፡ አልፎ አልፎ (4) አዎ፡ በጣም ጥቂት ጊዜ

(8) አላውቅም (9) ለመመለስ ፈቃደኛ አይደሉም

02120 |__| ጫት በሚጠቀሙበት ወቅት ሺሻ አጭሰው ነበር/ እያጨሱ ነው?

(0) አይደለም (1) አዎ፡ ሁልጊዜ (2) አዎ፡ በተደጋጋሚ

(3) አዎ፡ አልፎ አልፎ (4) አዎ፡ በጣም ጥቂት ጊዜ

(8) አላውቅም (9) ለመመለስ ፈቃደኛ አይደሉም

02121 |__| ጫት በሚጠቀሙበት ጊዜ ትንባሆ አኝከዋል/እያኙኩ ነበር?

(0) አይደለም (1) አዎ፡ ሁልጊዜ (2) አዎ፡ በተደጋጋሚ

(3) አዎ፡ አልፎ አልፎ (4) አዎ፡ በጣም ጥቂት ጊዜ

(8) አላውቅም (9) ለመመለስ ፈቃደኛ አይደሉም

02122 |__| የተደጋጋሚ የአፍ የጤና እክል አጋጥሞዎት ነበር/ እያጋጠመዎት ነው?

(0) አይደለም (1) አዎ፡ የቁስለ አይነት (2) አዎ፡ በአፍ ውስጥ (3)አዎ ሌላ

02123 በአብዛኛው የጤና ችግር ያጋጠመው የአፍ ክፍል ____________________

02124 ሌላ ችግር ካለ ____________________________________(ይግለፁልን)

ክፍል 3. አልኮል የመጠጣት ታሪክ

03001 |__| ቢያንስ በሳምንት አንድ ጊዜ ለስድስት ወራት በላይ የአልኮል (ጠላ፣ጠጅ፣አረቄ፣ ቢራ፣ እንደ ውስኪ ያሉ) መጠጦችን ጠጥተዋል?

(0) አይደለም (1) አዎ (8) አላውቅም (9) ለመመለስ ፈቃደኛ አይደሉም
(የተሳታፊው/ዋ ምላሽ "አይደለም" ከሆነ ጥያቄ ቁጥር 04001 ይሂዱ!)

03002 |__|__| በአማካይ በአንድ ሳምንት ውስጥ ምን ያህል (በቢራ ጠሩሙስ ፣ጠላ/ወይን /ውስኪ በብርጨጭቆ) የአልኮል መጠጥ ይጠጣሉ?

(እባክዎ የአልኮል መጠጫ እቃዎችን የሚያሳየውን ምስል ለተሳታፊው ያሳዩ!)

03003 |__|__| አልኮል መጠጣት የጀመሩት በስንት ዓመትዎ ነው?

03004 |__| በአሁኑ ሰዓት የአልኮል መጠጥ ተጠቃሚ ነዎት?

(0) አይደለም (1) አዎ (8) አላውቀውም (9) ለመመለስ ፈቃደኛ አይደሉም
ለቀድሞ የአልኮል ተጠቃሚዎች ብቻ የሚጠየቅ (የተሳታፊው/ዋ ምላሽለጥያቄ ቁጥር 03004 "አይደለም" ከሆነ)

03005 |__|__| አልኮል መጠጣት ካቆሙ ስንት ዓመት ሆነዎት?

ከዚህ በመቀጠል በሕይዎት ዘመንዎ ስለጠጧቸው የአልኮል መጠጦች እጠይቀዎታለሁ፡፡

ሀ. የአልኮል መጠጥ ጠጥተዎ ያውቃሉ______________?

ለ. የአልኮል መጠጥ መጠጣት የጀመሩት በስንት ዓመትዎ ነው______________?

ሐ. መጠጣት በጀመሩበት ጊዜ በሳምንት ምን ያክል መጠጥ ይጠጡ ነበር_______?

መ. የሚጠጡትን የአልኮል መጠጥ መጠን ይግለፁልን_______?

(እባክዎ የአልኮል መጠጫ እቃዎችን የሚያሳየውን ምስል ለተሳታፊው ያሳዩ!)

ሰ. የሚጠጡት መጠጥ የአልኮል መጠን በመቶኛ(%) ስንት ነው?**(የመጠጡ አይነት ሌላ "ሌላ" ከሚለው ምርጫ ውስጥ ከሆነ)**

ረ. መጠጥ መጠጣት አቁመዋል፣ ጨምረዋል ወይስ ቀንሰዋል? መልስዎ "አዎ" ከሆነ ካቆሙ ወይንም አጠጣጥዎን ከቀየሩ ስንት ጊዜ ሆነዎት?**(የመጠጥ አጠጣጥ ሁኔታ ከተቀየረ ከጥያቄ "ለ" ጀምሮ ይድገሙት)**

<table>
<tr><th></th><th>ሀ</th><th>ለ</th><th>ሐ</th><th>መ</th><th>ሰ</th><th>ረ</th></tr>
<tr><th>የአልኮል መጠጥ አይነት</th><th>(0) አይደለም
(1) አዎ</th><th>መጠጥ የጀመሩበት እድሜ</th><th>በሳምንት የሚጠጡት ጊዜ*</th><th>የእየንዳንዱ የሚጠጡት መጠን(ሚ.ሊ)</th><th>የአልኮል መጠን በመቶኛ</th><th>መጠጣት የቀጠሉበት እድሜ</th></tr>
<tr><td rowspan="5">ቢራ</td><td rowspan="5">03006 |__|</td><td>03007 |__|__|</td><td>03008 |__|__|</td><td>03009 |__|__|__|</td><td>03010____</td><td>03011 |__|__|__|</td></tr>
<tr><td>03012 |__|__|</td><td>03013 |__|__|</td><td>03014 |__|__|__|</td><td>03015____</td><td>03016 |__|__|__|</td></tr>
<tr><td>03017 |__|__|</td><td>03018 |__|__|</td><td>03019 |__|__|__|</td><td>03020____</td><td>03021 |__|__|__|</td></tr>
<tr><td>03022 |__|__|</td><td>03023 |__|__|</td><td>03024 |__|__|__|</td><td>03025____</td><td>03026 |__|__|__|</td></tr>
<tr><td>03027 |__|__|</td><td>03028 |__|__|</td><td>03029 |__|__|__|</td><td>03030____</td><td>03031 |__|__|__|</td></tr>
<tr><td rowspan="5">ጠላ</td><td rowspan="5">03032|__|</td><td>03033 |__|__|</td><td>03034 |__|__|</td><td>03035 |__|__|__|</td><td>03036____</td><td>03037 |__|__|__|</td></tr>
<tr><td>03038 |__|__|</td><td>03039 |__|__|</td><td>03040 |__|__|__|</td><td>03041____</td><td>03042 |__|__|__|</td></tr>
<tr><td>03043 |__|__|</td><td>03044 |__|__|</td><td>03045 |__|__|__|</td><td>03046____</td><td>03047 |__|__|__|</td></tr>
<tr><td>03048 |__|__|</td><td>03049 |__|__|</td><td>03050 |__|__|__|</td><td>03051____</td><td>03052 |__|__|__|</td></tr>
<tr><td>03053 |__|__|</td><td>03054 |__|__|</td><td>03055 |__|__|__|</td><td>03056____</td><td>03057 |__|__|__|</td></tr>
<tr><td rowspan="5">ወይን</td><td rowspan="5">03058|__|</td><td>03059 |__|__|</td><td>03060 |__|__|</td><td>03061 |__|__|__|</td><td>03062____</td><td>03063 |__|__|__|</td></tr>
<tr><td>03064 |__|__|</td><td>03065 |__|__|</td><td>03066 |__|__|__|</td><td>03067____</td><td>03068 |__|__|__|</td></tr>
<tr><td>03069 |__|__|</td><td>03070 |__|__|</td><td>03071 |__|__|__|</td><td>03072____</td><td>03073 |__|__|__|</td></tr>
<tr><td>03074 |__|__|</td><td>03075 |__|__|</td><td>03076 |__|__|__|</td><td>03077____</td><td>03078 |__|__|__|</td></tr>
<tr><td>03079 |__|__|</td><td>03080 |__|__|</td><td>03081 |__|__|__|</td><td>03082____</td><td>03083 |__|__|__|</td></tr>
<tr><td rowspan="4">ጠጅ</td><td rowspan="4">03084 |__|</td><td>03085 |__|__|</td><td>03086 |__|__|</td><td>03087 |__|__|__|</td><td>03088____</td><td>03089 |__|__|__|</td></tr>
<tr><td>03090 |__|__|</td><td>03091 |__|__|</td><td>03092 |__|__|__|</td><td>03093____</td><td>03094 |__|__|__|</td></tr>
<tr><td>03095 |__|__|</td><td>03096 |__|__|</td><td>03097 |__|__|__|</td><td>03098____</td><td>03099 |__|__|__|</td></tr>
<tr><td>03100 |__|__|</td><td>03101 |__|__|</td><td>03102 |__|__|__|</td><td>03103____</td><td>03104 |__|__|__|</td></tr>
</table>

	ሀ	ለ	ሐ	መ	ሰ	ረ
የአልኮል መጠጥ አይነት	(0) አይደለም (1) አዎ	መጠጥ የጀመሩበት እድሜ	በሳምንት የሚጠጡት ጊዜ*	የእየንዳንዱ የሚጠጡት መጠን(ሚ.ሊ)	የአልኮል መጠን በመቶኛ	መጠጣት የቀጠሉበት እድሜ
		03105 |__|__|	03106 |__|__|	03107 |__|__|__|	03108____	03109 |__|__|__|
ከባድ መጠጥ	03110 |__|	03111 |__|__|	03112 |__|__|	03113 |__|__|__|	03114____	03115 |__|__|__|
		03116 |__|__|	03117 |__|__|	03118 |__|__|__|	03119____	03120 |__|__|__|
		03121 |__|__|	03122 |__|__|	03123 |__|__|__|	03124____	03125 |__|__|__|
		03126 |__|__|	03127 |__|__|	03128 |__|__|__|	03129____	03130 |__|__|__|
		03131 |__|__|	03132 |__|__|	03133 |__|__|__|	03134____	03135 |__|__|__|
አረቄ	03136 |__|	03137 |__|__|	03138 |__|__|	03139 |__|__|__|	03140____	03141 |__|__|__|
		03142 |__|__|	03143 |__|__|	03144 |__|__|__|	03145____	03146 |__|__|__|
		03147 |__|__|	03148 |__|__|	03149 |__|__|__|	03150____	03151 |__|__|__|
		03152 |__|__|	03153 |__|__|	03154 |__|__|__|	03155____	03156 |__|__|__|
		03157 |__|__|	03158 |__|__|	03159 |__|__|__|	03160____	03161 |__|__|__|
ሌላ ካለ	03162 |__|					
በፅሁፍ__________ 03163		03164 |__|__|	03165|__|__|	03166 |__|__|__|	03167____	03168 |__|__|__|
በፅሁፍ__________ 03169		03170 |__|__|	03171 |__|__|	03172 |__|__|__|	03173____	03174 |__|__|__|

	ሀ	ለ	ሐ	መ	ሠ	ረ
የአልኮል መጠጥ አይነት	(0) አይደለም (1) አዎ	መጠጥ የጀመሩበት እድሜ	በሳምንት የሚጠጡት ጊዜ*	የእየንዳንዱ የሚጠጡት መጠን(ሚ.ሊ)	የአልኮል መጠን በመቶኛ	መጠጣት የቀጠሉበት እድሜ
በፅሁፍ_________ 03175		03176 |___|___|	03177 |___|___|	03178 |___|___|___|	03179_____	03180 |___|___|___|
በፅሁፍ_________ 03181		03182 |___|___|	03183 |___|___|	03184 |___|___|___|	03185_____	03186 |___|___|___|
በፅሁፍ_________ 03187		03188 |___|___|	03189 |___|___|	03190 |___|___|___|	03191_____	03192 |___|___|___|

የአልኮል መጠጦች   መጠጫ እቃዎች እስከ መጠናቸው

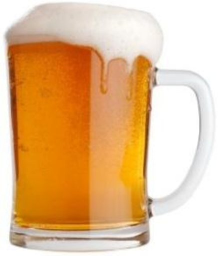	ቢራ/ድራፍት<br><br>- 285 ሚ.ሊ<br>- 570 ሚ.ሊ<br>- 1 ትንሽ ጠርሙስ/1 ትንሽ ቆርቆሮ (330 ሚ.ሊ)<br>- 1 ትልቅጠርሙስ/1 ትልቅቆርቆሮ (500 ሚ.ሊ)
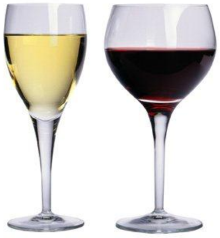	ወይን (ሻምፓኝ)<br><br>- 1 ትንሽ ብርጭቆ (125 ሚ.ሊ)<br>- 1 ትልቅ ብርጭቆ (250 ሚ.ሊ)<br>- 1 ጠርሙስ = 6 ትንሽ ብርጭቆ (750 ሚ.ሊ)
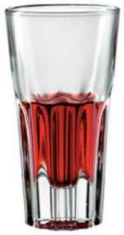	ከምግብ በፊት የሚጠጣ የአልኮል መጠጥ(ቬርሞዝ፣ማርቲኒ፣ ሲንዛኖ፣ ዲቦኔት፣ ፓስቲስ፣ ወዘተ.)<br><br>- 1 ነጠላ መለኪያ (25 ሚ.ሊ)<br>- 1 ጥንድ መለኪያ (50 ሚ.ሊ)<br>- 1 ጠርሙስ = 28 ነጠላመለኪያ (700 ሚ.ሊ)
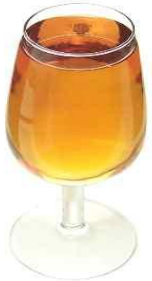	ከባድ አልኮል የያዘ ወይን(ሸሪ፣ ፖርት)<br><br>- 1 ትንሽ ብርጭቆ (50 ሚ.ሊ)
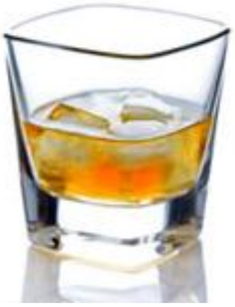	በማቅረር የሚዘጋጁ የአልኮል መጠጦች<br><br>- 1 ነጠላ መለኪያ (25 ሚ.ሊ)<br>- 1ትልቅ መለኪያ (35 ሚ.ሊ)<br>- 1 ጥንድ መለኪያ (50 ሚ.ሊ)<br>- 1 ጠርሙስ = 28 መለኪያ (700ሚ.ሊ)

የአልኮል መጠጥ በብዛት የሚጠጣበትን ክፍለ ጊዜ በተመለከተ

03193 |__| በአንድ ጊዜ አምስት መጠጦችን ጠጥተው ያውቃሉ?

(0) አይደለም (1) አዎ (8) አላውቅም (9) ለመመለስ ፈቃደኛ አይደሉም **(የተሳታፊው ምላሽአይደለም ከሆነ ወደ ጥያቄ ቁጥር 04001 ይሒዱ!)**

03194 |__|__| መልስዎ "አዎ" ከሆነ፣ በእየንዳንዱ በሚጠጡበት ሰዓት ምን ያህል

ይጠጣሉ?

03195 |__|__| በአመት በዚህ አይነት የመጠጥ ሁኔታ ለምን ያህል ጊዜ ይጠጣሉ?

03196 |__|__|__| ይህንን አይነት የመጠጣት ዘዴ የጀመሩት በስንት አመትዎ

ነው?

03197 |__|__| በዚህ አይነት የመጠጣት ዘዴ በዚህ አይነት የመጠጣት ዘዴ የቆዩት

ለምን ያህል ጊዜ ነው?

ክፍል 4. የሻይና ቡና የመጠጣት ታሪክ

ሻይ

04001 |__| ለስድስት ወር ወይንም ከዛ በላይ በሳምንት አንድ ስኒ ሻይ ጠጥተዋል?

(0) አይደለም (1) አዎ (8)አላውቅም (9) ለመመለስ ፈቃደኛ አይደሉም

(የተሳታፊው/ዋ ምላሽ "አይደለም" ከሆነ ወደ ጥያቄ ቁጥር 04013 ይሒዱ!)

04002 |__|__|__| ሻይ መጠጣት የጀመሩት በስንት ዓመትዎ ነው?

04003 |__|__|__| ለስንት ዓመት ያህል ሻይ ጠጥተዋል?

04004 |__|__|__| በአንድ ሳምንት ውስጥ በአማካኝ ምን ያህል ስኒ ሻይ ይጠጣሉ?

04005 |__|__|__|__| በአማካኝ ሻይ ለመጠጣት የሚጠቀሙበት ስኒ መጠኑ (በሚ.ሊ) ምን ያህል ነው?

(8888) አላውቀውም (9999) ለመመለስ ፈቃደኛ አይደሉም

የሚጠጡት ሻይ፡

04006 |__| ጥቁር ሻይ

(0) አይደለም (1) አዎ (8) አላውቅም (9) ለመመለስ ፈቃደኛ አይደሉም

04007 |__| አረንጓዴ ሻይ

0) አይደለም (1) አዎ (8) አላውቅም (9) ለመመለስ ፈቃደኛ አይደሉም

04008 |__| የአበባ ሻይ

0) አይደለም (1) አዎ (8) አላውቅም (9) ለመመለስ ፈቃደኛ አይደሉም

04009 |__| ለባህላዊ ህክምናና ለመአም ማጣፈጫ ከሚወሉ ቅጠሎች የሚፈላ ሻይ

0) አይደለም (1) አዎ (8) አላውቅም (9) ለመመለስ ፈቃደኛ አይደሉም

04010 |__| ሌላ

0) አይደለም (1) አዎ (8) አላውቅም (9) ለመመለስ ፈቃደኛ አይደሉም

04011 ይግለፁልን ______________________

04012 |__|መጠጣት የሚፈልጉት የሻይ አይነት የፈላ ወይንስ የቀዘቀዘ ነው?

(1) ቀዝቃዛ ሻይ (2) ሞቅ ያለ ሻይ (3) ትኩስ ሻይ (4) በጣም ትኩስ ሻይ

(8) አላውቅም (9) ለመመለስ ፈቃደኛ አይደሉም

<u>ቡና</u>

04013 |__| ለስድስት ወር ወይንም ከዛ በላይ በሳምንት አንድ ስኒ ቡና ጠጥተዋል?

(0) አይደለም (1) አዎ (8) አላውቅም (9) ለመመለስ ፈቃደኛ አይደሉም

(የተሳታፊው/ዋ ምላሽ "አይደለም" ከሆነ ወደ ጥያቄ ቁጥር 05001 ይሂዱ፡፡)

04014 |__|__|__| ዓመት፡ ቡና መጠጣት የጀመሩት በስንት ዓመትዎ ነው?

04015 |__|__|__| ለስንት ዓመት ያክል ቡና ጠጥተዋል?

04016 |__| ቡና የሚቆላው እቤትዎ ነው? (1) አይደለም (2) አዎ፣

04017 <u>መልስዎ "አዎ"</u> ከሆነ በሳምንት ውስጥ ምን ያክል ጊዜ ቡና ያፈላሉ? |__|__|__|

04018 |__| በተደጋጋሚ ቡናን የሚጠጡት ቤትዎ ውስጥ ነው?

(0) አይደለም (1) አዎ (8) አላውቅም (9) ለመመለስ ፈቃደኛ አይደሉም

04019 |__| በተደጋጋሚ ቡናን የሚጠጡት ቡና ቤት ወይም ሬስቶራንት ውስጥ ነው?

(0) አይደለም (1) አዎ (8) አላውቅም (9) ለመመለስ ፈቃደኛ አይደሉም

04020 |__| ቡና የሚጠጡበት ሌላ ቦታ አለ?

(0) አይደለም (1) አዎ (8) አላውቅም (9) ለመመለስ ፈቃደኛ አይደሉም

04021 |__| ሌላ ቡና የሚጠጡበት ቦታ ካለ ይግለፁልን__________________

04022 |__| ቡናን በተደጋጋሚ የሚጠጡት

(1) በስኳር ብቻ (2) በወተት/በክሬም ብቻ (3) በጨው

(4) በስኳርና በወተት (5) በጨውና በወተት (8)አላውቅም (9) ለመመለስ ፈቃደኛ አይደሉም

04023 |__| መጠጣት የሚፈልጉት የቡና አይነት፡-

(1) ከበድ ያለ ቡና (2) መካከለኛ ቡና (3) ቀለል ያለ ቡና

(4) እርግጠኛ አይደሉም (8)አላውቅም (9) ምላሽ ለመስጠት ፈቃደኛ አይደሉም

04024 |__|__|__| በአንድ ሳምንት ውስጥ ምን ያህል ስኒ ቡና ይጠጣሉ?

4.2025 |__|__|__|__| በአማካይ ቡና ለመጠጣት የተጠቀሙበት/ ሚጠቀሙበት ስኒ መጠኑ (በሚሊ ሊትር) ምን ያህል ነው?

(8888) አላውቀውም (9999) ለመመለስ ፈቃደኛ አይደሉም

ክፍል 5. የአመጋገብ ሁኔታን በተመለከተ

እባክዎ ከአንድ አመት በፊት የነበረውን የአመጋገብ ሁኔታ በማስታወስ ከዚህ ቀጥሎ ያሉትን ጥያቄዎች ይመልሱልን!(የላይኛው ስርዓተ- እንሽርሽሪት ካንሰር ላለባቸው የጥናቱ ተሳታፊዎች፤ ካንሰሩ በመምርመራ ከመታዎቁ አንድ መት በፊት ያለውን የአመጋገብ ሁኔታ)

05001 |__| በቤትዎ በቋሚነት የሚመገቡት የምግብ አይነት የትኛው ነው?

(1)እንጀራ (4) ሌላ ካለ (ይግለፁልን) (05002)__________________

(2) የበቆሎ ዳቦ (3) ቆጮ (8) አላውቅም (9) ለመመለስ ፈቃደኛ አይደሉም

05003 |__| ጠቅለል ባለ ሁኔታ ይመገቡት የነበረው የምግብ አይነት፡-

(1) በጣም ጨዋማ (2) ጨዋማ (3) ብዙም ጨዋማ ያልሆነ

(4) ጨዋማ ያልሆነ (8) አላውቀም (9) ለመመለስ ፈቃደኛ አይደሉም

05004 |__| ቢያንስ በወር አንድ ጊዜ ገንፎ ተመግበው ያውቃሉ?

(0) አይደለም (1) አዎ (8) አላውቅም (9) ለመመለስ ፈቃደኛ አይደሉም

(የተሳታፊው/ዋ ምላሽ "አይደለም" ከሆነ፤ እባክዎ ወደ ጥያቄ ቁጥር 05007 ይሒዱ)

05005 |__|__| መልስዎ "አዎ" ከሆነ በወር ውስጥ ስንት ጊዜ ገንፎ ይመገባሉ?

05006 ገንፎን የሚመገቡት እንዴት ነው ?

(1) በጣም በትኩሱ (2) በትኩሱ (3) ከቀዘቀዘ በኃላ

(8) አላውቅም (9) ለመመለስ ፈቃደኛ አይደሉም

05007 |__| ምግብ ሳይበላሽ ለማስቀመጥ ፍሪጅ ይጠቀማሉ?

(0) አይደለም (1) አዎ (8) አላውቅም (9) ለመመለስ ፈቃደኛ አይደሉም

(የተሳታፊው/ዋ ምላሽ "አይደለም" ከሆነ እባክዎ ወደ ጥያቄ ቁጥር 05009 ይሒዱ)

05008 |__|__| በህይወት ዘመንዎ ምን ያህል ጊዜ ምግብን ፍሪጅ ውስጥ

አስቀምጠዋል?

05009 |__|__| በቤትዎ ውስጥ ስንት ሰዎች ምግብ በአንድ ላይ ይመገባሉ?

<u>ከአንድ አመት በፊት የነበረ የምግብ አመጋገብ</u>
ከዚህ በታች የተዘረዘሩትን የምግብ አይነቶች ለምን ያህል ጊዜ ተመግበዋቸዋል?

<table>
<tr><th rowspan="2">የምግብ አይነት</th><th colspan="10">ባለፈው አንድ አመት የምግብ አመጋገብ ድግግሞሽ
(ለእያንዳንዱ የምግብ አይነቶች ከተሰጡት አማራጮች ውስጥ የሚመገቡትን ጊዜ ይግለፁልን)</th></tr>
<tr><th>3 ጊዜ በየቀን</th><th>2 ጊዜ በየቀን</th><th>1 ጊዜ በየቀን</th><th>5-6 ጊዜ በየሳምንቱ</th><th>3-4 ጊዜ በየሳምንቱ</th><th>1-2 ጊዜ በየሳምንቱ</th><th>በሳምንት ከአንደ ጊዜ ያነሰ</th><th>በጭራሽ</th><th>አላውቅም</th><th>ፈቃደኛ አይደሉም</th></tr>
<tr><td>05010 ስጋ (የበሬ፣ የላም፣ የበግ፣ የፍየል)</td><td>|__|$_1$</td><td>|__|$_2$</td><td>|__|$_3$</td><td>|__|$_4$</td><td>|__|$_5$</td><td>|__|$_6$</td><td>|__|$_7$</td><td>|__|$_8$</td><td>|__|$_9$</td><td>|__|$_{10}$</td></tr>
<tr><td>05011 የዶሮ ስጋ</td><td>|__|$_1$</td><td>|__|$_2$</td><td>|__|$_3$</td><td>|__|$_4$</td><td>|__|$_5$</td><td>|__|$_6$</td><td>|__|$_7$</td><td>|__|$_8$</td><td>|__|$_9$</td><td>|__|$_{10}$</td></tr>
<tr><td>05012 ዓሳ</td><td>|__|$_1$</td><td>|__|$_2$</td><td>|__|$_3$</td><td>|__|$_4$</td><td>|__|$_5$</td><td>|__|$_6$</td><td>|__|$_7$</td><td>|__|$_8$</td><td>|__|$_9$</td><td>|__|$_{10}$</td></tr>
<tr><td>05013 ወተትና የወተት ተዋጽኦ ውጤቶች</td><td>|__|$_1$</td><td>|__|$_2$</td><td>|__|$_3$</td><td>|__|$_4$</td><td>|__|$_5$</td><td>|__|$_6$</td><td>|__|$_7$</td><td>|__|$_8$</td><td>|__|$_9$</td><td>|__|$_{10}$</td></tr>
</table>

05014 እንቁላል	|__|1	|__|2	|__|3	|__|4	|__|5	|__|6	|__|7	|__|8	|__|9	|__|10
05015 አረንጓዴ አትክልቶች	|__|1	|__|2	|__|3	|__|4	|__|5	|__|6	|__|7	|__|8	|__|9	|__|10
05016 ሌሎች አትክልቶች	|__|1	|__|2	|__|3	|__|4	|__|5	|__|6	|__|7	|__|8	|__|9	|__|10
05017 ፍራፍሬ	|__|1	|__|2	|__|3	|__|4	|__|5	|__|6	|__|7	|__|8	|__|9	|__|10
05018 ባቄላና የባቄላ ውጤቶች	|__|1	|__|2	|__|3	|__|4	|__|5	|__|6	|__|7	|__|8	|__|9	|__|10
05019 የታሸጉ/የተብላሉ ምግቦች	|__|1	|__|2	|__|3	|__|4	|__|5	|__|6	|__|7	|__|8	|__|9	|__|10

ክፍል 6. የአፍ ጤንነትን በተመለከተ

06001 |__| ጥርስዎትን ምን ያህል ጊዜ ያፀዱታል?

(0) በጭራሽ (1) በሳምንት ከአንድ ጊዜ ያነሰ (2) በሳምንት ከአንድ እስከ ሁለት ጊዜ (3) አንድ አንድ ቀን እያለፍኩ (4) በቀን አንድ ጊዜ (5) በቀን ሁለት ጊዜ (6) በቀን ሶስት ጊዜ (7) በቀን ከ3 ጊዜ በላይ

(8) አላውቅም (9) ለመመለስ ፈቃደኛ አይደሉም

06002 |__|__| በአሁኑ ሰዓት ምን ያህል ጥርስ አለዎት?

(የጥናቱ ተሳታፊ የጥርሳቸውን ቁጥር የማያውቁት ከሆነ እባክዎ ቃለመጠይቁን

የሚያካሔዱት ሰው ተሳታፊው/ዋ ጥርሳቸውን ይቆጥሩ ዘንድ ፈቃድ በመጠየቅ ይርዷቸው)

06003 |__| የሰው ሰራሽ ጥርስ አድርገዋል?

(0) አይደለም (1) አዎ (8) አላውቅም (9) ለመመለስ ፈቃደኛ አይደሉም

(የተሳታፊው/ዋ ምላሽ "አይደለም" ከሆነ እባክዎ ወደ ጥየቄ ቁጥር 06005 ይሂዱ!)

06004 |__|__|__| የሰው ሰራሽ ጥርስ ማድረግ የጀመሩት ከስንት አመትዎ ጀምሮ ነው?

06005 |__| የጥርስ ሀኪም ጋር ምን ያህል ጊዜ ታይተዋል?

(0) በጭራሽ (1) አንድ ጊዜ ከአምስት ዓመት በላይ (2) ከሁለት እስከ አራት ዓመት ውስጥ አንድ ጊዜ (3) በዓመት አንድ ጊዜ (4) በዓመት ከአንድ ጊዜ በላይ (8) አላውቅም (9) ለመመለስ ፈቃደኛ አይደሉም

ክፍል 7. የተለያ በሽታዎች ታሪክን በተመለከተ

ከዚህ በመቀጠል ቀድሞ አምዎት ስለነበረ የጤና እክል እጠይቀዎታለሁ?

ሀ. ከዚህ በመቀጠል ስለተዘረዘሩት የጤና እክሎች ሃኪም ነግሮዎታል?

ለ. መልስዎ "አዎ" ከሆነ ይህ የጤና ችግር እንዳለብዎት የተነገረዎት በስንት አመትዎ ነው?

ሐ. ለችግሩ ህክምና አግኝተው ነበር?

መ. ህክምናው ምን ነበር ?

ሠ. ለሕመምዎ የቀዶ ጥገና ሕክምና ተደርጎልዎት ነበር?

(ሀ) የቀድሞ ህመም<br><br>(0) አይደለም<br>(1) አዎ<br>(8) አላውቅም<br>(9) ፈቃደኛ አይደሉም	(ለ) እድሜ በአመት<br><br>888 መልሱ አላውቀውም ከሆነ	(ሐ) ሕክምና<br><br>(0) አይደለም<br>(1) አዎ<br>(8) አላውቅም<br>(9)ፈቃደኛ አይደሉም	(መ) ህክምናው በፅሁፍ ይቀመጥ ወይም ተገቢ ከሆን አላውቀውም	(ሠ) የቀዶ ጥገና ህክምና<br><br>(0) አይደለም<br>(1) አዎ<br>(8) አላውቅም<br>(9) ፈቃደኛ አይደሉም
07001 I__I ከፍተኛ የደም ግፊት	[07002]<br>I__I__I__I	[07003] I__I	[07004]	[07005] I__I
07006 I__I የስኳር በሽታ (በእርግዝና ወቅት የሚከሰተውን አያጠቃልልም)	[07007]<br>I__I__I__I	[07008] I__I	[07009]	[07010] I__I
07011 I__I ቲቢ	[07012]<br>I__I__I__I	[07013] I__I	[07014]	[07015] I__I
07016 I__I ተደጋጋሚ የጥርስ መድማት	[07017]<br>I__I__I__I	[07018] I__I	[07019]	[07020] I__I
07021 I__I አፍ ውስጥ የሚታዩ ነጫጫ ነገሮች /Oral leukoplakia	[07022]<br>I__I__I__I	[07023] I__I	[07024]	[07025] I__I
07026 I__I የአፍ የመገናኛ አካል ለምፅ/Oral submucous fibrosis (OSF)	[07027]<br>I__I__I__I	[07028] I__I	[07029]	[07030] I__I
07031 I__I ድብርት	[07032]<br>I__I__I__I	[07033] I__I	[07034]	[07035] I__I

07036 |__| አስም	[07037] |__|__|__|	[07038] |__|	[07039]	[07040] |__|
07041 |__| አለርጅ	[07042] |__|__|__|	[07043] |__|	[07044]	[07045] |__|

ክፍል 8. የቤተሰብ የካንሰር ታሪክ

እባክዎ ያልዎትን ወንድም፣ እህት ፣ ሴት እና ወንድ ልጅ ይንገሩኝ፡፡(የእንጀራ እህትንና የእንጀራ ወንድምን አያጠቃልልም)

08001 ወንድም |__|__| 08002 እህት |__|__|

08003 ወንድ ልጅ |__|__| 08004 ሴት ልጅ |__|__|

08005 ከእርስዎ የመጀመሪያ ደረጃ ዘመድ(ከላይ ከተጠቀሱት ውስጥ) ካንሰር ያለበት ሰው አለ?

(0) አይደለም (1) አዎ (8) አላውቅም (9) ለመመለስ ፈቃደኛ አይደሉም

(የተሳታፊው/ዋ መልስ " አየደለም " ከሆነ ወደ ጥያቄ ቁጥር 09001 ይሒዱ!)

እባክዎ የእርስዎን የመጀመሪያ ደረጃ ዘመድ ካንሰር ያለበት ሰው ይዘርዝሩልን፡፡

(1) አባት (2) እናት (3) ወንድም (4)እህት (5)ወንድ ልጅ (6) ሴት ልጅ

ዘመድ (ኮድ 1-6)	[08006] |__|	[08013] |__|	[08020] |__|	[08027] |__|	[08034] |__|	[08041] |__|	[08048] |__|
ካንሰር ያለበት ቦታ (በፅሁፍ)	[08007]	[08014]	[08021]	[08028]	[08035]	[08042]	[08049]
ካንሰሩ መኖሩ የታወቀበት እድሜ	[08008] |__|__|	[08015] |__|__|	[08022] |__|__|	[08029] |__|__|	[08036] |__|__|	[08043] |__|__|	[08050] |__|__|
ካንሰር ያለበት	**(እባክዎ ኮዱን ከቃለ መጠይቀ በኃላ ይሙሉት(ለጥናቱ አስተባባሪ))**						
	[08009]	[08016]	[08023]	[08030]	[08037]	[08044]	[08051]

<table>
<tr><td>ቦታ(ICD-9)</td><td>|__|__|__|</td><td>|__|__|__|</td><td>|__|__|__|</td><td>|__|__|__|</td><td>|__|__|__|</td><td>|__|__|__|</td><td>|__|__|__|</td></tr>
<tr><td>ካንሰር ያለበት ቦታ (በፅሁፍ)</td><td>[08010]</td><td>[08017]</td><td>[08024]</td><td>[08031]</td><td>[08038]</td><td>[08045]</td><td>[08052]</td></tr>
<tr><td>ካንሰሩ መኖሩ የታወቀበት እድሜ</td><td>[08011]
|__|__|</td><td>[08018]
|__|__|</td><td>[08025]
|__|__|</td><td>[08032]
|__|__|</td><td>[08040]
|__|__|</td><td>[08046]
|__|__|</td><td>[08053]
|__|__|</td></tr>
<tr><td rowspan="2">ካንሰር ያለበት ቦታ (ICD-9)</td><td colspan="7">(እባክዎ ኮዱን ከቃለ መጠይቀ በኃላ ይሙሉት(ለጥናቱ አስተባባሪ))</td></tr>
<tr><td>[08012]
|__|__|__|</td><td>[08019]
|__|__|__|</td><td>[08026]
|__|__|__|</td><td>[08033]
|__|__|__|</td><td>[8.40]
|__|__|__|</td><td>[08047]
|__|__|__|</td><td>[08054]
|__|__|__|</td></tr>
<tr><td colspan="8">*እባክዎ ተጨማሪ ሰንጠረዥ ካስፈለገ ይጨምሩበት!</td></tr>
</table>

ክፍል 9፡ የስራ ታሪክ

09001 |__|__|__| ሙሉ ሰዓት (በሳምንት ከ 20 ሰዓት በላይ) መስራት የጀመሩት በስንት ዓመትዎ ነው?

09002 |__| በአሁኑ ሰዓት በመስራት ላይ ነዎት?

(0) አይደለም (1) አዎ (8) አላውቅም (9) ለመመለስ ፈቃደኛ አይደሉም

(የተሳታፊው/ዋ ምላሽ "አዎ" ከሆነ ወደ ጥያቄ ቁጥር "10001" ይሒዱ)

09003 |__|__|__| በአሁኑ ሰዓት በመስራት ላይ ካልሆኑ፣ ሙሉ ሰዓት (በሳምንት ከ 20 ሰዓት በላይ) መስራት ያቆሙት በስንት አመትዎ ነው?

09004 |__|__|__| በአጠቃላይ ለምን ያህል አመት በስራ አለም ቆዩ?

ስራ መስራት ከጀመሩበት እስከ ቅርብ ጊዜ በመስራት ላይ እስከሰሩት ስራ ድረስ

ቢያንስ ለአንድ ዓመት ስለሰሩት ስራ ይንገሩኝ?

ሀ. ስራ የጀመሩት በስንት አመትዎ ነበር?

ለ. ስራ መስራት ያቆሙት በስንት አመትዎ ነበር?

ሐ. የስራዎ መጠርያ ምን ነበር?

መ. ለሰሩት ስራ ኢንዱስትሪው ምን ነበር?

<table>
<tr><th>(ሀ) ከ
(እድሜ)</th><th>(ለ) እስከ
(እድሜ)</th><th>(ሐ) ስራ
(በፅሁፍ ያስቀምጡና ኮዱን ይጠቀሙ)</th><th>(መ) ኢንዱስትሪ
(በፅሁፍ ያስቀምጡና ኮዱን ይጠቀሙ)</th><th>ኮድ</th></tr>
<tr><td rowspan="2">09005|__|__|</td><td rowspan="2">09006|__|__|</td><td>09007|__|__|</td><td>09009 |__|__|</td><td rowspan="10">የስራው ኮድ: (ISCO 1968)
1. የሞያ፣ የጉልበት እና ተመሳሳይ ስራ
2. አስተዳደር ስራ
3. የፅህፈትና ተመሳሳያ ስራ
4. የሽያጭ ስራ
5. የአገልግሎት ስራ
6. የግብርና/እርሻ፣ እንሰሳ የማድለብና ተክል የመንከባከብ ስራ፣ አሳ የማጥመድና የአደን ስራ
7. የማምረትና ተመሳሳይ ስራ
8. የመጓጓዣ መሳሪያዎች ጥገናና የጉልበት ስራ
9. ውትድርና
10. ሌላ
የኢንደስተሪ ኮድ: (ISIC)
1. ግብርና/እርሻ፣ አደን፣ ተክል እንክብካቤና ዓሣ ማጥመድ
2. ማዕድን ማውጣትና ቁፋሮ ስራ
3. ፋብሪካ የማምረት ስራ
4. ኤልክትሪክ፣የጋዝና የውሃ ስራ
5. ግንባታ
6. የጅምላና የችርቻሮ
7. ምግብ ቤትና ሆቴል
8. ማከማቸትና መጓጓዝ
9. የመረጃ ግንኙነት
10. የፋይናንስ፣ የዋስትና, የቦት ስራ እና የቢዝነስ አገልግሎቶች</td></tr>
<tr><td>09008</td><td>09010</td></tr>
<tr><td rowspan="2">09011|__|__|</td><td rowspan="2">09012|__|__|</td><td>09013|__|__|</td><td>09015|__|__|</td></tr>
<tr><td>09014</td><td>09016</td></tr>
<tr><td rowspan="2">09017|__|__|</td><td rowspan="2">09018|__|__|</td><td>09019|__|__|</td><td>09021|__|__|</td></tr>
<tr><td>09020</td><td>09022</td></tr>
<tr><td rowspan="2">09023|__|__|</td><td rowspan="2">09024|__|__|</td><td>09025|__|__|</td><td>09029 |__|__|</td></tr>
<tr><td>09026</td><td>09028</td></tr>
<tr><td rowspan="2">09029|__|__|</td><td rowspan="2">09030|__|__|</td><td>09031|__|__|</td><td>09033|__|__|</td></tr>
<tr><td>09032</td><td>09034</td></tr>
</table>

<table>
<tr><th>(ሀ) ከ
(እድሜ)</th><th>(ለ) እስከ
(እድሜ)</th><th>(ሐ) ስራ
(በፅሁፍ ያስቀምጡና ኮዱን ይጠቀሙ)</th><th>(መ) ኢንዱስትሪ
(በፅሁፍ ያስቀምጡና ኮዱን ይጠቀሙ)</th><th>ኮድ</th></tr>
<tr><td>09035|__|__|</td><td>09036|__|__|</td><td>09037|__|__|
____
09038</td><td>09039|__|__|
____
09040</td><td rowspan="5">11. የማህበረሰብ እና የግለሰብ አገልግሎት
12. ትምህርት
13. ሌላ</td></tr>
<tr><td>09041|__|__|</td><td>09042|__|__|</td><td>09043|__|__|
________0
9044</td><td>09045|__|__|
________09
046</td></tr>
<tr><td>09047|__|__|</td><td>09048|__|__|</td><td>09049|__|__|
________0
9050</td><td>09051|__|__|
________09
052</td></tr>
<tr><td>09053|__|__|</td><td>09054|__|__|</td><td>09055|__|__|
________0
9056</td><td>09057|__|__|
________09
058</td></tr>
<tr><td>09059|__|__|</td><td>09060|__|__|</td><td>09061|__|__|
________0
9062</td><td>09063|__|__|
________09
064</td></tr>
</table>

*እባክዎ ተጨማሪ ሰንጠረዥ ካስፈለገ ይጨምሩበት!

ክፍል 10፡ የመኖሪያ አካባቢ ታሪክ

እባክዎ ከተወለዱበት አመት ጀምሮ እስከ አሁን ድርስ ቢያንስ ለአንድ ዓመት የኖሩበትን አካባቢ ይንገሩኝ? (የቆዩበትን ሰዓት የፈለገውን ያህል ቢሆን የተወለዱበት ቦታንና አሁን የሚኖሩበትን የመኖሪያ አካባቢ ይንገሩኝ)

ሀ. በየትኛው ከተማና ሀገር ይኖሩ ነበር?

ለ. በዚህ ከተማ መኖር የጀመሩት ከሰንት አመትዎ ጀምሮ ነው?

ሐ. ወደሌላ የመኖሪያ አካባቢ የሔዱት በስንት አመትዎ ነው?

መ. የከተማዋ ስፋት እንደ ከተማ ወይንስ እንደ ገጠር ነው የምትቆጠረው?

ሰ. በአካባቢው <u>በዋናነት ምግብ የሚነበሰልበት ዘዴ</u> ምንድን ነው?

ረ. ምግብ የሚሰራበት ዘዴ ምን ያህል ጭስ አለው?

ሰ) ምግብ የማብሰያ ዘዴ	
1=የተፈጥሮ ጋዝ	6=ኤሌክትሮማግኔቲክ ኦቨን
2=ኤልክትሪክ	7=ጋዝ(ፕሮፔን)
3= የከሰል ምድጃ	8=በጣም ጥቂት ጊዜ ምግብቤት ውስጥ ማብሰል
4=የእንጨት ምድጃ	9= ሌላ
5=ማይክሮዌብ ኦቨን*	88=አላውቅም
99=ለመመለስ ፈቃደኛ አይደሉም	

ረ) ቤት ውስጥ የጭስ መጠን
0=ጭስ የለም,
1=በጣም ትንሽ ጭስ
2= የተወሰነ ጭስ
3=ብዙ ጭስ

(ሀ)ከተማ እና ክልል(ሃገር፡ ከኢትጵያ ውጭ ከሆነ (መልሱን በፅሁፍ ያስቀምጡ)	(ለ) ከ (እድሜ)	(ሐ) እስከ (እድሜ)	(መ) የከተማ መጠን (0) ከተማ (1) ገጠር (8) አላውቅም (9) ፈቃደኛ አይደሉም	(ሰ) ምግብ የማብሰያ መንገድ	(ረ) ቤት ውስጥ የጭስ መጠን	(ሠ) በቤትዎ ውስጥ የምግብ ማብሰያ ማድቤት አለ?
[10001-10002]	[10003] |__|__|	[10004] |__|__|	[10005] |__|	[10006] |__|	[10007] |__|	[10008] |__|

(ሀ)ከተማ እና ክልል(ሃገር፡ ከኢትጵያ ውጭ ከሆነ (መልሱን በፅሁፍ ያስቀምጡ)	(ለ) ከ (እድሜ)	(ሐ) እስከ (እድሜ)	(መ) የከተማ መጠን (0) ከተማ (1) ገጠር (8) አላውቅም (9) ፈቃደኛ አይደሉም	(ሰ) ምግብ የማብሰያ መንገድ	(ረ) ቤት ውስጥ የጭስ መጠን	(ሠ) በቤትዎ ውስጥ የምግብ ማብሰያ ማድቤት አለ?
[10009-10010]	[10011] |__|__|	[10012] |__|__|	[10013] |__|	[10014] |__|	[10015] |__|	[10016] |__|
[10017-10018]	[10019] |__|__|	[10020] |__|__|	[10021] |__|	[10022] |__|	[10023] |__|	[10024] |__|
[10025-10026]	[10027] |__|__|	[10028] |__|__|	[10029] |__|	[10030] |__|	[10031] |__|	[10032] |__|
[10033-10034]	[10035] |__|__|	[10036] |__|__|	[10037] |__|	[10038] |__|	[10039] |__|	[10040] |__|

(ሀ)ከተማ እና ክልል(ሃገር፡ ከኢትጵያ ውጭ ከሆነ (መልሱን በፅሁፍ ያስቀምጡ)	(ለ) ከ (እድሜ)	(ሐ) እስከ (እድሜ)	(መ) የከተማ መጠን (0) ከተማ (1) ገጠር (8) አላውቅም (9) ፈቃደኛ አይደሉም	(ሰ) ምግብ የማብሰያ መንገድ	(ረ) ቤት ውስጥ የጭስ መጠን	(ሠ) በቤትዎ ውስጥ የምግብ ማብሰያ ማድቤት አለ?
[10041-10042]	[10043] |__|__|	[10044] |__|__|	[10045] |__|	[10046] |__|	[10047] |__|	[10048] |__|
[10049- 10050]	[10051] |__|__|	[10052] |__|__|	[10053] |__|	[10054] |__|	[10055] |__|	[10056] |__|
[10057- 10058]	[10059] |__|__|	[10060] |__|__|	[10061] |__|	[10062] |__|	[10063] |__|	[10064] |__|
[10065- 10066]	[10067] |__|__|	[10068] |__|__|	[10069] |__|	[10070] |__|	[10071] |__|	[10072] |__|
	[10075] |__|__|	[10076] |__|__|	[10077] |__|	[10078] |__|	[10079] |__|	[10008] |__|

(ሀ)ከተማ እና ክልል(ሃገር፡ ከኢትጵያ ውጭ ከሆነ (መልሱን በፅሁፍ ያስቀምጡ)	(ለ) ከ (እድሜ)	(ሐ) እስከ (እድሜ)	(መ) የከተማ መጠን (0) ከተማ (1) ገጠር (8) አላውቅም (9) ፈቃደኛ አይደሉም	(ሰ) ምግብ የማብሰያ መንገድ	(ረ) ቤት ውስጥ የጭስ መጠን	(ሥ) በቤትዎ ውስጥ የምግብ ማብሰያ ማድቤት አለ?
[10073- 10074]						

*እባክዎ ተጨማሪ ሰንጠረዥ ካስፈለገ ይጨምሩበት!

ቃለ መጠይቁ እዚህ ላይ ያበቃል፡፡ ጊዜን መስዋዕት አድረገው ጥያቄዎቹን ስለመለሰልን ከልብ እናመሰግናለን!

ስለ ቃለ መጠይቅ አድራጊው/ዋ

(እባክዎ ከዚህ ቀጥሎ ያሉትን ጥያቄዎች ልክ የጥናቱ ተሳታፊ ካመሰገኑ በኃላ ወዲያዉኑ ይሙሉት!)

(011001) የቃለ መጠይቅ አድራጊው/ዋ ስም፡---

(011002) ቃለ መጠይቁ የተጠናቀቀበት (ቀን/ወር/ዓ.ም)

|__|__|/|__|__| / |__|__|__|__|

(011003) ቃለ መጠይቁ የተጠናቀቀበት ሰዓት |__|__|: |__|__|(በ12 ሰዓት አቆጣጠር)

(011004) ቃለ መጠይቁ ስንት ሰዓት ወሰደ? |__|__| ሰዓት: |__|__| ደቂቃ

(011005) |__| ቃለ መጠይቁ በሚደረግበት በማንኛውም ክፍል ከእርስዎና ከጥናቱ ተሳታፊ ውጭ ሌላ ሰው ነበር?

(0)አይደለም (1) አዎ (8) አላውቅም (9) ለመመለስ ፈቃደኛ አይደሉም

(011006) መልስዎ " አዎ" ከሆነ የነበረው ሰው ማን ነው?

(0)እናት (2) አባት (3) እህት (4) ወንድም (5) ሴት ልጅ
(6) ወንድ ልጅ (7) ቅድመ አያቶች (8) በለቤት (9) ጊደኛ

(10) የሆስፒታል ሰራተኛ (11) ሌላ

(011007) ቃለ መጠይቁ የተካሔደው ከአምርኛ ቋንቋ ውጭ በሌላ ቋንቋ ነው?

(0) አይደለም (1) አዎ (8) አላውቅም (9) ለመመለስ ፈቃደኛ አይደሉም

(011008) መልስዎ አዎ ከሆነ በምን ቋንቋ ነው የተካሔደው?______________________(በፅሁፍ)

(011009) ትርጉሙን ያካሔደው ማን ነው? ______________________(በፅሁፍ)

(011010) |__| የትብብር ሁኔታ፡ 0=ደካማ 1=ደህና 2=ጥሩ

(011011) ስለ ቃለ መጠይቁ ተጨማሪ አስተያየት(በፅሁፍ ይግለፁት)

____

____

____
