# Supplementary material for: Qat use and esophageal cancer in Ethiopia: A pilot case-control study
Source: PLoS One. 2017 Jun 8;12(6):e0178911. doi: 10.1371/journal.pone.0178911 (PMC5464578; doi:10.1371/journal.pone.0178911)
Supplement: S1 Table — (DOCX) [file pone.0178911.s004.docx]

|  |  |  | Cases | | | | | | Matched Controls | | | | | |
| --- | --- | --- | --- | --- | --- | --- | --- | --- | --- | --- | --- | --- | --- | --- |
|  |  |  | Esophageal | | Junction | | Total Cases | | Inpatient | | Healthy | | Total Controls | |
|  |  |  | n=61 | | n=12 | | n=73 | | n=40 | | n=93 | | n=133 | |
|  | Qat use | Never use qat | 39 | 64% | 8 | 67% | 47 | 64% | 30 | 75% | 69 | 74% | 99 | 74% |
|  |  | Ever | 22 | 36% | 4 | 33% | 26 | 36% | 10 | 25% | 24 | 26% | 34 | 26% |
|  |  |  |  |  |  |  |  |  |  |  |  |  |  |  |
|  | Qat use, duration | Never use qat | 39 | 64% | 8 | 67% | 47 | 64.4% | 30 | 75% | 69 | 74.2% | 99 | 74.4% |
|  | (years) | < 30 | 12 | 20% | 2 | 16.7% | 14 | 19% | 5 | 12.5% | 8 | 8.6% | 13 | 9.8% |
|  |  | ≥ 30 | 10 | 16.4% | 2 | 16.7% | 12 | 16.4% | 5 | 12.5% | 14 | 15.0% | 19 | 14.3% |
|  |  | Unknown |  |  |  |  |  |  |  |  | 2 | 2.2% | 2 | 1.5% |
|  |  |  |  |  |  |  |  |  |  |  |  |  |  |  |
|  | Qat use, frequency days/week | Never use qat | 39 | 64% | 8 | 67% | 47 | 64.4% | 30 | 75% | 69 | 74.2% | 99 | 74.4% |
|  |  | Less than daily | 9 | 14.7% | 0 |  | 9 | 12.3% | 4 | 10% | 10 | 10.7% | 14 | 10.5% |
|  |  | Daily | 12 | 19.7% | 3 | 25% | 15 | 20.5% | 6 | 15% | 14 | 15% | 20 | 15% |
|  |  | Don't know | 1 | 1.6% | 1 | 8.3% | 2 | 2.7% |  |  |  |  |  |  |
|  |  |  |  |  |  |  |  |  |  |  |  |  |  |  |
|  | Qat use, sessions / day | Never use qat | 39 | 64% | 8 | 67% | 47 | 64.4% | 30 | 75% | 69 | 74.2% | 99 | 74.4% |
|  |  | 1 | 16 | 26.2% | 1 | 8.3% | 17 | 23.3% | 6 | 15% | 18 | 19.4% | 24 | 18% |
|  |  | 2+ | 6 | 9.8% | 2 | 16.7% | 8 | 11.0% | 4 | 10% | 6 | 6.5% | 10 | 7.5% |
|  |  | Don't know |  |  | 1 | 8.3% | 1 | 1% |  |  |  |  |  |  |
|  |  |  |  |  |  |  |  |  |  |  |  |  |  |  |
|  | Duration Qat in mouth | Median (hours) | 4 |  | 3 |  | 4 |  | 4 |  | 4 |  | 4 |  |
|  |  | N | 20 |  | 3 |  |  |  | 10 |  | 20 |  |  |  |
|  |  |  |  |  |  |  |  |  |  |  |  |  |  |  |
|  | Qat wad kept overnight | Never use qat | 39 | 64% | 8 | 67% | 47 | 64% | 30 | 75% | 69 | 74.2% | 99 | 74.4% |
|  |  | No | 13 | 21.3% | 2 | 16.7% | 15 | 20.5% | 6 | 15% | 16 | 17.2% | 22 | 16.5% |
|  |  | Yes | 6 | 9.8% | 2 | 16.7% | 8 | 11% | 0 |  | 3 | 3.2% | 3 | 2.3% |
|  |  | Missing | 3 | 4.9% |  |  | 3 | 4.1% | 4 | 10% | 5 | 3.2% | 9 | 6.8% |
|  |  |  |  |  |  |  |  |  |  |  |  |  |  |  |
|  | Qat part chewed | Never use qat | 39 | 64% | 8 | 67% | 47 | 64% | 30 | 75% | 69 | 74.2% | 99 | 74.4% |
|  |  | Fresh tender leaves | 16 | 26.2% | 2 | 16.7% | 18 | 24.6% | 6 | 15.0% | 15 | 16.1% | 21 | 15.8% |
|  |  | Fresh stems | 5 | 8.2% | 2 | 16.7% | 7 | 9.6% | 3 | 7.5% | 4 | 4.3% | 7 | 5.2% |
|  |  | Both leaves, stems | 1 | 1.6% |  |  | 1 | 1.4% | 1 | 2.5% | 2 | 2.2% | 3 | 2.3% |
|  |  | Missing |  |  |  |  |  |  |  |  | 3 | 3.2% | 3 | 2.3% |
|  |  |  |  |  |  |  |  |  |  |  |  |  |  |  |
|  | Qat leaves hand-cleaned | Never use qat | 39 | 64% | 8 | 67% | 47 | 64% | 30 | 75% | 69 | 74.2% | 99 | 74.4% |
|  |  | Yes | 6 | 10% | 2 | 16.7% | 8 | 11% | 9 | 23% | 18 | 19% | 27 | 20% |
|  |  | No | 16 | 26% | 2 | 17.0% | 18 | 25% | 1 | 3% | 5 | 5% | 6 | 5% |
|  |  | Missing |  |  |  |  |  |  |  |  | 1 | 1% | 1 | 0.7% |
|  |  |  |  |  |  |  |  |  |  |  |  |  |  |  |
|  | Smoke cigarettes in Qat session | Never use qat | 39 | 64% | 8 | 67% | 47 | 64% | 30 | 75% | 69 | 74.2% | 99 | 74.4% |
|  |  | No | 11 | 18% | 3 | 25% | 14 | 19.2% | 5 | 12.5% | 12 | 12.9% | 17 | 12.8% |
|  |  | Yes, always or usually | 11 | 18% | 1 | 8.3% | 12 | 16.4% | 5 | 12.5% | 10 | 10.8% | 15 | 11.3% |
|  |  | Missing |  |  |  |  |  |  |  |  | 2 |  | 2 | 1.5% |
|  |  |  |  |  |  |  |  |  |  |  |  |  |  |  |
|  | Any tobacco use | Never | 43 | 70% | 7 | 58% | 50 | 68% | 31 | 78% | 78 | 84% | 109 | 82% |
|  |  | Ever | 18 | 30% | 5 | 42% | 23 | 32% | 9 | 23% | 15 | 16% | 24 | 18% |
|  |  |  |  |  |  |  |  |  |  |  |  |  |  |  |
|  | Ever cigarette smoke | No | 48 | 79% | 9 | 75% | 57 | 78% | 32 | 80% | 78 | 84% | 110 | 83% |
|  |  | Yes | 13 | 21% | 3 | 25% | 16 | 22% | 8 | 20% | 15 | 16% | 23 | 17% |
|  |  |  |  |  |  |  |  |  |  |  |  |  |  |  |
|  | Ever water-pipe smoke | No | 60 | 98% | 12 | 100% | 72 | 98.6% | 40 | 100% | 93 | 100% | 132 | 99% |
|  |  | Yes | 1 | 2% | 0 |  | 1 | 1.4% | 0 |  | 0 |  | 1 | 1% |
|  |  |  |  |  |  |  |  |  |  |  |  |  |  |  |
|  | Ever pipe smoke | No | 61 | 100% | 11 | 91.7% | 72 | 98.6% | 39 | 97.5% | 93 | 100% | 132 | 99% |
|  |  | Yes | 0 |  | 1 | 8.3% | 1 | 1.4% | 1 | 2.5% | 0 |  | 1 | 1% |
|  |  |  |  |  |  |  |  |  |  |  |  |  |  |  |
|  | Ever smokeless tobacco | No | 56 | 92% | 9 | 75% | 65 | 89% | 39 | 98% | 93 | 100% | 132 | 99% |
|  |  | Yes | 5 | 8% | 3 | 25% | 8 | 11% | 1 | 2% | 0 |  | 1 | 1% |
|  |  |  |  |  |  |  |  |  |  |  |  |  |  |  |
|  | Ever user of Qat and/or tobacco | None | 33 | 54% | 5 | 42% | 38 | 52% | 27 | 68% | 66 | 71% | 93 | 70% |
|  |  | Only qat | 10 | 16% | 2 | 17% | 12 | 16% | 4 | 10% | 12 | 13% | 16 | 12% |
|  |  | Only tobacco | 6 | 10% | 3 | 25% | 9 | 12% | 3 | 8% | 3 | 3% | 6 | 5% |
|  |  | Both | 12 | 20% | 2 | 17% | 14 | 19% | 6 | 15% | 12 | 13% | 18 | 14% |
