# Supplementary material for: Qat use and esophageal cancer in Ethiopia: A pilot case-control study
Source: PLoS One. 2017 Jun 8;12(6):e0178911. doi: 10.1371/journal.pone.0178911 (PMC5464578; doi:10.1371/journal.pone.0178911)
Supplement: S2 Table — (DOCX) [file pone.0178911.s005.docx]

| Lifestyle factors | | Cases | | | | | | Matched Controls | | | | | |
| --- | --- | --- | --- | --- | --- | --- | --- | --- | --- | --- | --- | --- | --- |
|  |  | Esophageal | | Junction | | Total | | Inpatient | | Healthy | | Total | |
|  |  | n=61 | | n=12 | | n=73 | | n=40 | | n=93 | | n=133 | |
| Alcohol use | Never | 43 | 70% | 8 | 67% | 51 | 69.9% | 27 | 68% | 52 | 56% | 79 | 59.4% |
|  | Ever | 18 | 30% | 4 | 33% | 22 | 30.1% | 13 | 32% | 41 | 44% | 54 | 40.6% |
|  |  |  |  |  |  |  |  |  |  |  |  |  |  |
| Alcohol type | Never | 43 | 70% | 8 | 67% | 51 | 69.9% | 27 | 68% | 52 | 56% | 79 | 59.4% |
|  | Ever excluding Aräqe | 9 | 15% | 3 | 25% | 12 | 16.4% | 10 | 25% | 28 | 30% | 38 | 28.5% |
|  | Ever including Aräqe | 9 | 15% | 1 | 8% | 10 | 13.6% | 3 | 7% | 13 | 14% | 16 | 12.0% |
|  |  |  |  |  |  |  |  |  |  |  |  |  |  |
| Staple food | Enjera | 35 | 57% | 5 | 42% | 40 | 54.8% | 26 | 65% | 67 | 72% | 93 | 69.9% |
|  | Kocho | 11 | 18% | 5 | 42% | 16 | 21.9% | 6 | 15% | 10 | 11% | 16 | 12.0% |
|  | Corn bread | 8 | 13% | 1 | 8% | 9 | 12.3% | 2 | 5% | 12 | 13% | 14 | 10.5% |
|  | Other | 7 | 11% |  |  | 7 | 9.6% | 5 | 12.5% | 4 | 4% | 9 | 6.8% |
|  | Unknown |  |  | 1 | 8% | 1 | 1.4% | 1 | 2.5% |  |  | 1 | 0.8% |
|  |  |  |  |  |  |  |  |  |  |  |  |  |  |
| Porridge consumption | No | 17 | 28% | 4 | 33% | 21 | 29% | 14 | 35% | 33 | 35% | 47 | 35% |
|  | Yes | 44 | 72% | 8 | 67% | 52 | 71% | 25 | 63% | 60 | 65% | 85 | 64% |
|  | Unknown |  |  |  |  |  |  | 1 | 2% |  |  | 1 | 1% |
|  |  |  |  |  |  |  |  |  |  |  |  |  |  |
| Green leafy | Never | 11 | 18% | 2 | 17% | 13 | 17.8% |  |  | 4 | 4% | 4 | 3% |
| vegetables | Less than weekly | 19 | 31% | 2 | 17% | 21 | 28.8% | 7 | 18% | 8 | 9% | 15 | 11.3% |
| consumption | 1-6 times per week | 10 | 16% | 1 | 8% | 11 | 15.0% | 15 | 38% | 47 | 50.5% | 62 | 46.6% |
|  | Daily | 20 | 33% | 7 | 58% | 27 | 37% | 18 | 45% | 34 | 36.5% | 52 | 39.1% |
|  | Unknown | 1 | 2% |  |  | 1 | 1.4% |  |  |  |  |  |  |
|  |  |  |  |  |  |  |  |  |  |  |  |  |  |
| Other | Never | 15 | 24.5% | 4 | 33% | 19 | 26% | 1 | 2.5% | 5 | 5% | 6 | 4.5% |
| vegetables | Less than weekly | 16 | 26% | 3 | 25% | 19 | 26% | 7 | 7.5% | 7 | 7.5% | 14 | 10.5% |
| consumption | 1-6 times per week | 9 | 15% | 1 | 8% | 10 | 13.7% | 15 | 37.5% | 51 | 55% | 66 | 49.6% |
|  | Daily | 18 | 29.5% | 4 | 33% | 22 | 30.1% | 17 | 42.5% | 29 | 31% | 46 | 34.6% |
|  | Unknown | 3 | 4% |  |  | 3 | 4.1% |  |  | 1 | 1% | 1 | 0.8% |
|  |  |  |  |  |  |  |  |  |  |  |  |  |  |
| Fruit | Never | 13 | 21% | 6 | 50% | 19 | 26% | 14 | 35% | 32 | 34.4% | 46 | 34.6% |
| consumption | Less than weekly | 26 | 43% | 3 | 25% | 29 | 39.7% | 10 | 25% | 23 | 25% | 33 | 24.8% |
|  | 1-6 times per week | 15 | 24.5% | 1 | 8% | 16 | 21.9% | 8 | 20% | 27 | 29% | 35 | 26.3% |
|  | Daily | 5 | 8% | 2 | 17% | 7 | 9.6% | 2 | 5% | 4 | 4% | 6 | 4.5% |
|  | Unknown | 2 | 3% |  |  | 2 | 2.7% | 6 | 15% | 7 | 7.5% | 13 | 9.8% |
|  |  |  |  |  |  |  |  |  |  |  |  |  |  |
| Saltiness | Very salty | 17 | 28% | 2 | 17% | 19 | 26% |  |  | 2 | 2% | 2 | 1.5% |
| of food | Salty | 28 | 46% | 6 | 50% | 34 | 46.6% | 6 | 15% | 18 | 19% | 24 | 18% |
|  | Not salty/not very salty | 16 | 26% | 4 | 33% | 20 | 27.4% | 32 | 80% | 72 | 77% | 104 | 78.2% |
|  | Unknown |  |  |  |  |  |  | 2 | 5% | 1 | 1% | 3 | 2.3% |
|  |  |  |  |  |  |  |  |  |  |  |  |  |  |
| Smokiness | A lot of smoke | 34 | 56% | 9 | 75% | 43 | 58.9% | 17 | 42.5% | 38 | 41% | 55 | 41.3% |
| in home | Some smoke | 18 | 30% |  |  | 18 | 24.7% | 13 | 32.5% | 34 | 36.5% | 47 | 35.3% |
|  | No smoke/very little | 9 | 15% | 3 | 25% | 12 | 16.4% | 10 | 25% | 21 | 22.5% | 31 | 23.3% |
